# Detection of pharmacolipidodynamic effects following the intravenous and oral administration of gefitinib to C57Bl/6JRj mice by rapid UHPLC-MS analysis of plasma

**DOI:** 10.1038/s41598-024-66764-w

**Published:** 2024-07-24

**Authors:** Robert S. Plumb, Lee A. Gethings, Giorgis Isaac, Nyasha C. Munjoma, Ian D. Wilson

**Affiliations:** 1 Waters Corporation, Milford, MA 01757 USA; 2 Waters Corporation, Stamford Ave, Wilmslow, SK9 4AX UK; 3 Program in Molecular Medicine, University of Massachusetts, Chan Medical School, 373 Plantation Street, Worcester, MA 01605 USA; 4 Computational & Systems Medicine, Department of Metabolism, Digestion and Reproduction, Imperial College, Burlington Danes Building, Du Cane Road, London, W12 0NN UK

**Keywords:** Cancer, Drug discovery, Biomarkers

## Abstract

Omics-based biomarker technologies, including metabolic profiling (metabolomics/metabonomics) and lipidomics, are making a significant impact on disease understanding, drug development, and translational research. A wide range of patho-physiological processes involve lipids and monitoring changes in lipid abundance can give valuable insights into mechanisms of drug action, off target pharmacology and toxicity. Here we report changes, detected by untargeted LC–MS, in the plasma lipid profiles of male C57Bl/6JRj mice following the PO and IV administration of the epidermal growth factor receptor (EGFR) inhibitor gefitinib. Statistical analysis of the data obtained for both the IV and PO samples showed time-related changes in the amounts of lipids from several different classes. The largest effects were associated with a rapid onset of these changes following gefitinib administration followed by a gradual return by 24 h post dose to the type of lipid profile seen in predose samples. Investigation of the lipids responsible for the variance observed in the data showed that the PI, PC, LPC, PE and TG were subject to the largest disruption with both transient increases and decreases in relative amounts seen in response to administration of the drug. The pattern of the changes in the relative abundances of those lipids subject to variation appeared to be correlated to the pharmacokinetics of gefitinib (and its major metabolites). These observations support the concept of a distinct pharmacolipidodynamic relationship between drug exposure and plasma lipid abundance.

## Introduction

Gefitinib, N-(3-chloro-4-fluorophenyl)-7-methoxy-6-(3-morpholino-propoxy) quinazolin-4-amine (see Figure S1 for structure) is a drug approved in 2003 for the treatment of certain breast and non-small cell lung cancers, as well as some other specific cancers, under the brand name Iressa®^1–3^. Gefitinib, acts by interrupting epidermal growth signalling in target cancer cells in the tyrosine kinase domain and is classified as an epidermal growth factor receptor (EGFR) inhibitor. This protein is part of a family of ErbB receptors which include Her1 (EGFR), Her2 (erb-B2), Her3 (erb-B3) and Her4 (erb-B4), which are overexpressed in certain human cancers including those of the lung and breast^2^. This overexpression leads to activation of the apoptotic Ras (Rat sarcoma virus) signalling cascade resulting in excessive, unrestrained, cell replication. Due to its mechanism of action gefitinib is only effective against cancers which are overactive or have a mutated form of EGFR^4^.

Pharmacokinetic (PK) studies in animals and male humans have demonstrated that gefitinib is rapidly absorbed with good bioavailability^5^. However, gefitinib undergoes extensive biotransformation in preclinical species^5–10^ and humans (e.g.,^6,11^) resulting in a large number of drug metabolites. Previous in vivo and in vitro^12–15^ studies investigating the metabolism of gefitinib have shown that a diverse group of biotransformations are involved. Gefitinib thus undergoes oxidative metabolism of the morpholine ring, O-demethylation, and oxidative defluorination (e.g.,^6,11–15^) (mediated via the cytochrome (CYP) P450 enzymes CYP3A4 and 3A5, with contributions from CYP2D6^13,14^) followed, in some cases, by the further biotransformation of some of these functionalized metabolites to sulfate and glucuronide conjugates^9–17^. However, whilst the metabolic fate of the drug is now well characterized, in a number of species, the effect of gefitinib (and many other tyrosine kinase inhibitors (TKI)) on endogenous metabolism remains poorly understood (e.g. see^18^).

In our previous metabolomic analysis of urine, obtained following the IV administration of gefitinib to the C57Bl/6JRj mouse, we observed significant changes to their metabolic phenotypes^19^. These changes included e.g., increases in the relative concentrations of tryptophan, taurocholic acid, and the dipeptide lysyl-arginine as well as a decrease in the relative amounts of deoxyguanosine, 8-hydroxydeoxyguanosine, and asparaginyl-histidine in dosed compared to control animals. These increases and reductions in metabolite concentration showed both correlation and anticorrelation with the plasma pharmacokinetics and urinary excretion profiles of gefitinib and its metabolites leading to the conclusion that, by analogy to pharmacodynamic effects of the drug, these changes effectively represented “pharmacometabodynamic” changes^19^. However, because individual urine collections are taken over periods of several hours their analysis represents a relatively “blunt instrument” for studying responses to dynamic changes in the composition of plasma. Therefore, given the evidence that effects on lipid pathways (as well as glycolysis, TCA and amino acid metabolism) have been noted in response to TKI inhibitors (reviewed in^18^), and the well-known importance of lipid metabolism in cancer, we were prompted to investigate the response of the mouse plasma lipidome following gefitinib administration. Here we report the lipidomic analysis of mouse plasma following the IV and PO administration of gefitinib to the C57Bl/6JRj mouse.

## Results

The samples analysed here were those previously obtained following the IV and PO administration of gefitinib to male C57Bl/6JRj mice (10 and 50 mg/kg respectively) with a control group receiving vehicle only^9^ as summarized in Materials and Methods. Comparison of the IV and PO plasma PK data obtained in this earlier analysis confirmed that the drug was well absorbed by this species, with 53% oral bioavailability in these mice^9^. Mean peak observed plasma concentrations of 4.4 and 7.0 μg/mL of gefitinib for IV and PO administration respectively (Figures S2-S3) were obtained, and 15 circulating metabolites of the drug were also detected^9^.

### Lipidomic LC–MS/MS analysis of plasma

Plasma sample extracts were analysed using ultra high-performance liquid chromatography coupled to time of flight mass spectrometry (UHPLC-MS/MS) operated in reversed-phase (RP) gradient mode using a short (50 mm) vacuum jacketed column. In this system the lipid peaks were well resolved with the lysophospholipids (lysophosphatidylcholines (LPC), lysophosphatidylserines (LPS)) and free fatty acids eluting early in the gradient (0.5–1.2 min). Other polar lipids such as the phosphocholines (PC), phosphatidylethanolamines (PE) sphingomyelins (SM) and ceramides (Cer) eluted between 2.0–4.2 min and the mono-, di- and triglycerides (TG) eluted with the cholesterol esters between 4.3–5.2 min. Representative +ve and −ve electrospray ionization (ESI) mass chromatograms for the pooled QC samples are given in Figures S4 and S5. Analysis of all the QC data showed that there was no discernible analytical drift in the dataset, whilst the technical replicates showed good reproducibility in both +ve and −ve ESI. A total of 3576 and 2369 ions having a CV < 30% were detected in +ve and −ve ion modes respectively. Following unsupervised multivariate statistical analysis (MVA) by principal components analysis (PCA) with pareto scaling, the data were searched using publicly available databases to annotate the statistically significant lipids identified in the data set. Further, supervised, MVA using orthogonal partial least squares discriminant analysis (OPLS-DA) was then used to perform pairwise comparisons between time points of interest in the IV and PO data sets (the outcomes of these analyses are described below).

### Plasma lipidomic profiles of vehicle-dosed mice

Examination of the PCA data for the samples obtained from the −ve ESI MS data from the vehicle-only dosed mice showed some evidence for a time-related trajectory. Thus the 1 and 3 h samples moved away from the predose samples in the score plot before returning to a position similar to the predose samples at 24 h post vehicle administration. This behaviour suggests that what was being observed was diurnal variation as shown in Figure S6 where Pc 1 and Pc 2 accounted for 34.6 and 23.9% of the total variation detected in these data. A similar pattern of diurnal variation was observed for the +ve ESI data with the resulting PCA shown in Figure S7, (Pc 1 and Pc2 accounted for 30.4 and 21.3% of the total variation observed respectively). With both the −ve and +ve ESI PCA analysis there was also some evidence of separation of the data by cage of origin of the samples into two groups over the whole-time course of the study.

### Plasma lipidomic profiles of IV administered gefitinib mice

**Figure 1:**
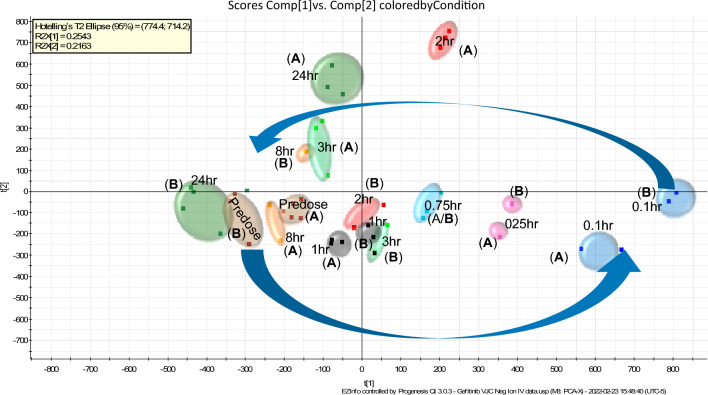
PCA of the −ve ESI LC/MS data for plasma obtained from male C57Bl6 mice following the IV administration of gefitinib at 10 mg/kg for the periods of predose, 0.1, 0.25, 0.5, 0.75, 1, 2, 3, 6, 8 and 24 h. These data were obtained following triplicate analysis of plasma samples. Animal cages are identified using the notation (**A**) and (**B**). The results show a clear time related trajectory in the data, from predose to 0.1 h with the subsequent time points showing a trend towards returning to the predose position which seems complete by 24 h.

Statistical analysis of the plasma lipid profiles obtained following IV administration revealed a clear time-related trajectory in response to gefitinib treatment in PCA. Further analysis of these data by PCA following removal of drug related signals (as described in Materials and methods), showed that Pc1 and Pc 2 accounted for 53.9% and 15.7% of the observed variation in the +ve ESI data respectively Figure S8, whilst for the −ve ESI data PC 1 and PC 2 accounted for 25.4 and 21.6% of the variation respectively (Fig. 1). The time-related trajectory observed in the −ve ESI data showed a rapid response to drug treatment with the 6 min (0.1 h) samples clustering furthest away from the predose samples. Over the subsequent 24 h time period the samples gradually returned towards the position of the predose samples, Fig. 1. The maximum observed separation of the samples from the predose position at 0.1 h post dose also coincided with the C_max_ of gefitinib and its major circulating metabolites (O-desmethyl-gefitinib and M605211; see ref^9^ and Figure S2) in these animals. However, as the LC–MS signals from gefitinib and its metabolites had been excluded from the statistical analysis, and therefore were not responsible for the observed variation, it seems most likely that the observed trajectory resulted from the pharmacological effects of the administration of the drug. Such a result strongly implies a pharmacolipidodynamic effect, as reported for the polar metabolites excreted in the urine of these mice^19^. It is interesting to note that by 24 h, when gefitinib and its metabolites were no longer detectable in plasma, lipid profiles had essentially returned to their “normal” predose values. The beginning of this return to “normal” from the maximum response at 0.1 h suggests a rapid and dynamic response to the fall in drug concentrations in the plasma of these IV dosed mice.

There was also some evidence of a “cage effect” in the results obtained for these samples (see Fig. 1), illustrated by the data for the predose, 2, 3 and 24 h samples, where the ellipses for the individual cages have been labelled A and B to indicate the two different metabowls.

The +ve ESI LC–MS data did not show as distinct a time-related trajectory as the −ve ESI LC–MS data, although there was a discernible pattern with the 0.1 h sample being furthest away from the predose samples in statistical space and the 0.25 h to 3 h samples grouped more closely together. However, the +ve ion data were different enough from that obtained for the vehicle-only samples to suggest effects related to the administration of the drug. Again, there was evidence of cage effects from the +ve ESI data as illustrated via the predose, 8 h and 24 h samples (Figure S8).

### Plasma lipidomic profiles of PO administered gefitinib mice

**Figure 2:**
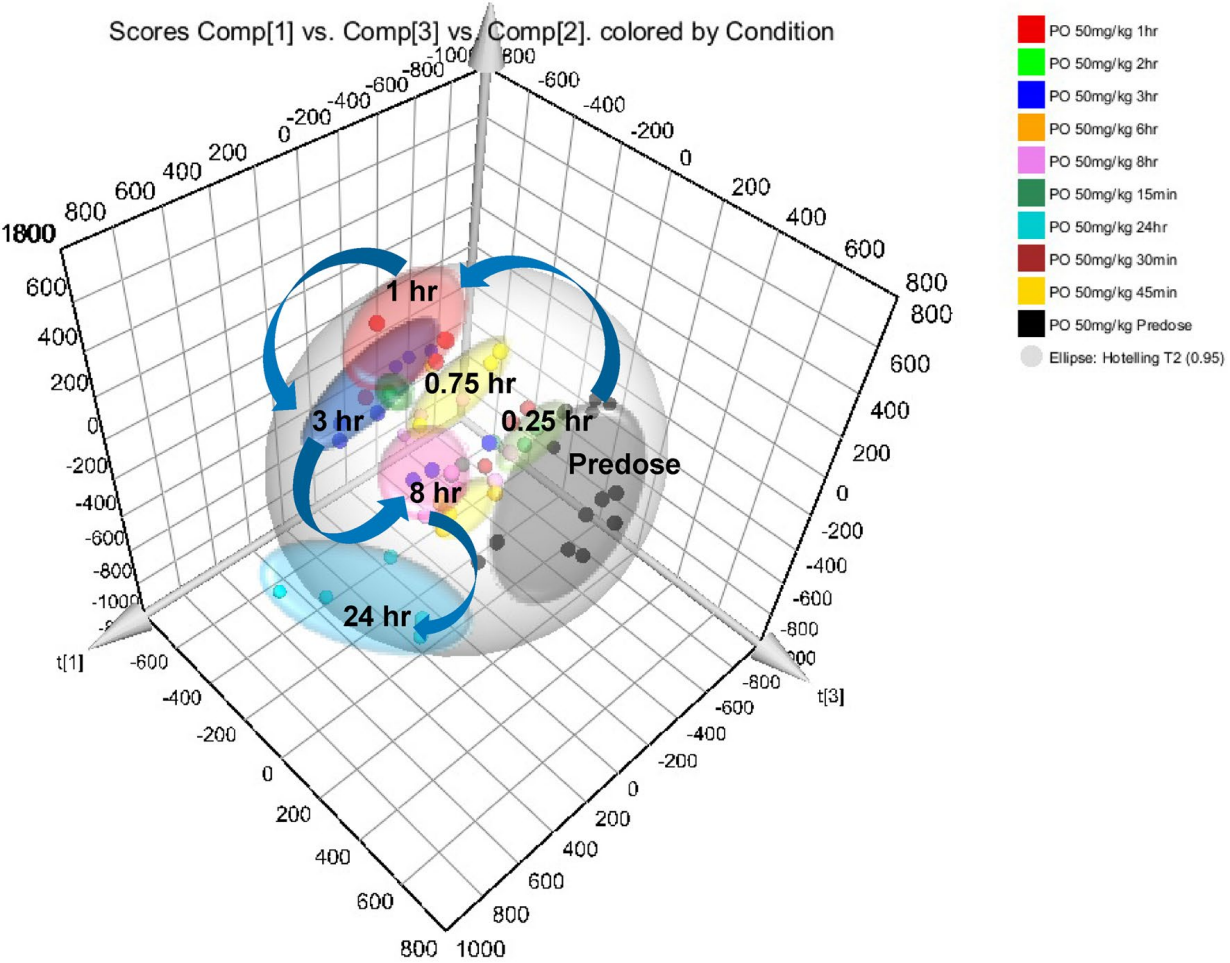
PCA of the −ve ESI LC–MS data for plasma obtained from male C57Bl6 mice following the PO administration of gefitinib at 50 mg/kg for the periods of predose, 0.25, 0.5, 0.75, 1, 2, 3, 6, 8 and 24 h. These data were obtained following triplicate analysis of plasma samples. The results show a clear time related trajectory in the data, from predose to 1 h with the 24 h samples still clearly separated from the predose samples.

PCA of the −ve ESI LC–MS data for the PO-dosed mice showed that PC 1 and PC 2 accounted for 28.3 and 19.3% of variation observed in these data (Figure S9). There was a clear trajectory in these data, moving away from the predose samples through the early time points, with the 1 h and 3 h time points being the furthest distance away from the predose (Fig. 2). As with the IV data the maximum variation from the predose samples was observed at a time point which matched the T_max_ of gefitinib in the PO pharmacokinetic profile (Figure S3)^9^. However, unlike the profile observed for the IV route, in the case of PO administration, neither the +ve or −ve ESI profiles had returned to a similar position in the statistical analysis to that occupied by the predose samples by 24 h post dose. As there were still low, but detectable, quantities of the drug and its metabolites present in the plasma 24 h post dose^9^ it is possible that these were still exerting a pharmacological effect on these mice, and that this was reflected in the lipidomic profile determined here.

PCA of the +ve ESI data showed that the PO samples produced a similar but not identical trajectory to that obtained with the IV samples (Figure S10) with PC 1 and PC 2 accounting for 30.7 and 22.8% of the variance in the data respectively. As seen for the IV route the dose-time-related effects were less apparent with the +ve ESI PO data than for the −ve ESI PO data. However, the 3 and 8 h data clustered the furthest distance from the predose and early time point samples, this is exemplified in the PC 1 vs PC 2 vs PC3 plot shown in Figure S11. As also observed for the −ve ESI PO data, the +ve ESI PCA score plot for the PO data showed that the samples had not returned to the predose condition at 24 h post dose. Similar to the IV samples there was some evidence for cage effects in the PO +ve ESI data, which were most pronounced for the predose, 1 h, and 8 h samples.

The unsupervised MVA analysis of the plasma sample data showed that the largest variance was observed in the −ve ESI data for both the IV and PO samples. In the IV sample set the 0.1 h timepoint showed the greatest separation from both the predose and 24 h samples. In the case of the PO samples the 1 h and 3 h samples, which corresponded to the highest observed plasma concentrations of gefitinib, were the ones that showed the greatest separation from the predose and 24 h samples. In an attempt to identify the lipids contributing most significantly to the observed variance in these data sets the IV predose and 0.1 and 0.25 h samples as well as the PO predose and 1 and 3 h samples were separately investigated using Orthogonal Projections to Latent Structures Discriminant Analysis (OPLS-DA). The results of the OPLS-DA of the −ve ESI data obtained following IV dosing of gefitinib, comparing the predose vs the 0.1 and 0.25 h sample data (analysed after removal of ions related to gefitinib) are presented in Figure S12A (with the data analysed including gefitinib-related signals shown in S12B for comparison). The signals related to components which had increased in relative abundance following dosing are contained in the upper right region of the S-plot (shaded red) and those whose relative abundance was higher in the predose samples are given in the lower left region (shaded in blue), the components are annotated with their individual *m/z* values (each component comprises a t_R_ and *m/z* pair, however, for clarity only the *m/z* value is shown). Similarly results for the equivalent analysis of the +ve ESI data from the PO predose vs 1 + 3 h samples are given in Figure S13, and the predose vs 3 h samples in Figure S14 (displayed in the same way as the IV data). Again, the components of interest are identified only by their *m/z* values.

### Lipid identification

Examples of the identification some of the dysregulated lipid t_R_ and *m/z* pairs from the −ve ESI of both the IV and PO sample analysis, filtered to remove lipids with a precursor mass measurement error greater than ± 5 ppm, are listed in Tables 1 and 2. Similarly, examples of the dysregulated lipids identified using +ve ESI following the IV and PO administration of gefitinib are provided in Tables S1 and S2. The lipids are reported as total number of carbon atoms and number of double bonds, as it was not possible from these data to identify the individual acyl chain length and location of the unsaturation. The lipids were provisionally identified via an initial search of databases and, where possible confirmed by comparing the accurate mass MS1 and MS2 data obtained from the plasma analysis with the corresponding MS1 and MS2 data of authentic lipid standards. These data, summarised in Tables 1 and 2, indicated that the lipids which showed an increase in relative abundance were dominated by the PE as well as phosphatidylinositol (PI) classes. Of the 12 identified lipids seen as showing a reduced relative abundance following either IV or PO dosing most came from the PI and PE. However, lipids identified as from the PE were also in the majority of the lipids that were increased in this sample set. The +ve ion data are summarized in Tables S1 and S2 show examples of TG, PC and LPC, most of which showed a relative increase in abundance.

**Table 1:** Summary of plasma lipid changes following PO administration of gefitinib (50 mg/kg) to male C57Bl6 mice (−ve ESI data).

t_R_ (min)_*m/z*	Compound ID	Mass error (ppm)	Isotope similarity	Max fold change	Increased abundance 1–3 h Post dose	Decreased abundance 1–3 h Post dose
1.91_808.5326	PS(34:0)	−2.6	93.8	3.1		x
0.32_498.2842	LPE(16:0)	1.0	89.6	1.7		x
2.39_911.5652	PI(40:5)	−0.3	85.1	1.6		x
1.96_859.5318	PI(36:3)	−2.8	87.0	1.6		x
0.74_582.3759	LPE(22:0)	−3.3	98.4	1.4		x
1.73_800.5430	PC (34:3)	−2.3	96.6	1.4	x	
3.27_844.6042	PE(40:2)	−3.8	97.4	1.3		x
2.22_861.5485	PI(36:2)	−1.6	98.2	1.3		x
2.61_887.5638	PI(38:3)	−1.9	98.5	1.2		x
1.58_857.5187	PI(36:4)	0.2	99.3	1.2		x
1.97_790.5389	PE(40:6)	−0.4	97.2	1.2	x	
1.97_850.5609	PE(41:6)	0.6	97.2	1.2	x

**Table 2:** Summary of plasma lipid changes following IV administration of gefitinib (10 mg/kg) to male C57B16 mice (−ve ESI data).

t_R_ (min)_*m/z*	Compound ID	Mass error (ppm)	Isotope similarity	Max fold change	Increased abundance 0.25 h Post dose	Decreased abundance 0.25 h Post dose
2.25_742.5388	PE(36:2)	0.0	94.3	1.6	x	
1.97_790.5384	PE(40:6)	−0.4	97.2	1.5		x
1.96_859.5318	PE(36:3)	−2.8	87.0	1.5		x
3.27_844.6042	PE(40:2)	−3.8	97.4	1.5		x
2.43_768.5539	PE(38:3)	−2.5	90.1	1.4		x

As previously indicated, the +ve ESI data did not show the clear time-related trajectory observed with the −ve ESI data however, there was an obvious separation between the predose, early time points and 6–8 h time points in the IV samples. To further investigate the effect of PO administration of gefitinib on the lipidome of the male mice MVA was used to compare the vehicle only and PO administration samples at the predose, 3, 8 and 24 h time points. These timepoints represented the drug free state, the time points at which greatest variation was observed, and the plasma concentration of gefitinib in the final sample of the study.

**Figure 3:**
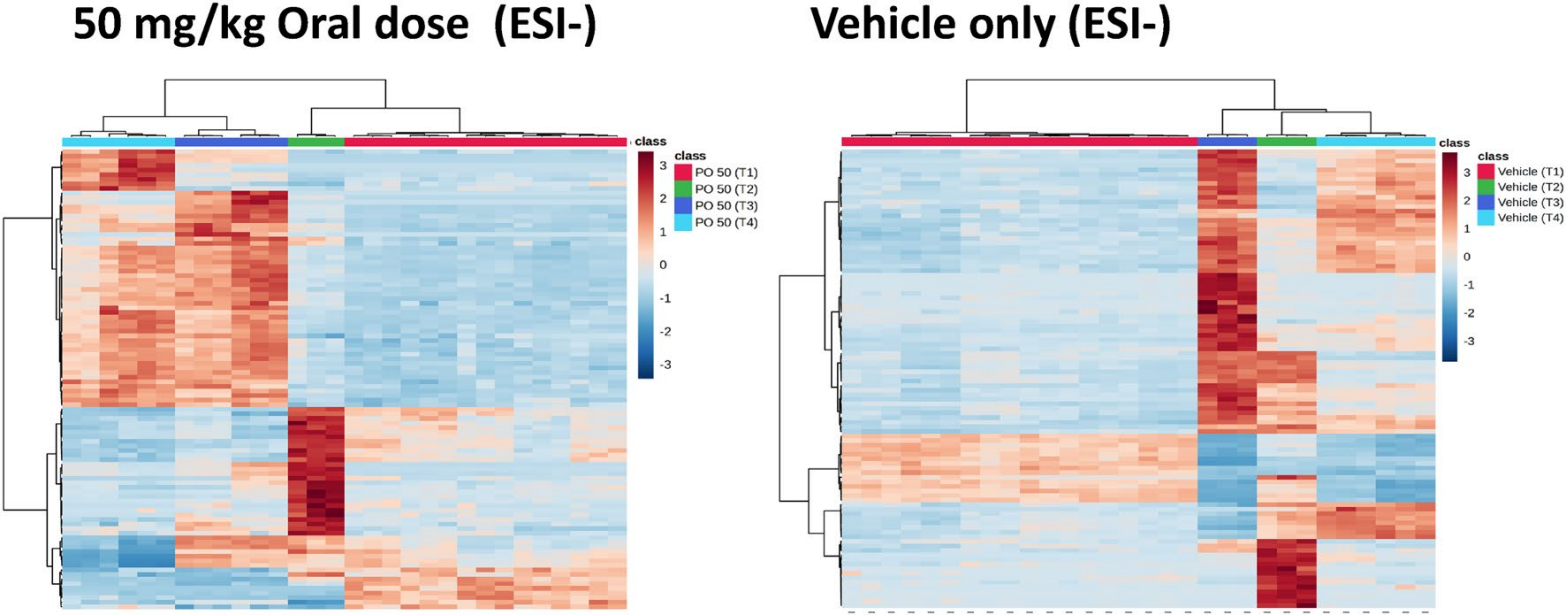
Heatmaps constructed using features consistent with the top 100 T-test/ANOVA values from the 50 mg/kg PO dose −ve ESI data. T1 = Predose, T2 = 3-h, T3 = 8-h and T4 = 24-h post dose. These heatmaps were produced using the top 100 t_R_–m/z 100 features identified by OPLS-DA as being responsible for differences between the plasma profiles of gefitinib dosed and control animals. These features are listed (for both +ve and −ve ESI) in the supplementary data (Table S4).

Following filtering to remove the gefitinib and metabolite signals, the +ve ESI and −ve ESI data for the IV and PO dose routes were transferred to MetaboAnalyst and subjected to OPLS-DA. Heatmaps were then constructed using features for the top 100 T-test/ANOVA values followed by hierarchical clustering (including at the sample level) using Ward clustering and Euclidean distance. The heatmaps produced for the top 100 lipids identified by MVA as significant detected in the −ve ESI of the PO samples are given in Fig. 3. The predose vehicle-only mouse samples all showed a similar lipid profile but, following vehicle administration a small change was observed in the lipid profiles of the top 100 lipids and by the 3 h time point there was a distinct increase in the expression of these lipids. The 8 h samples then showed a reduction in lipid expression with samples returning to a similar profile to the predose samples by the 24 h time point. The PO 50 mg/kg predose samples also exhibited, as would be expected, a similar pattern of lipid expression for the top 100 lipids as that of the vehicle only predose samples. However, by 3 h post dose the samples obtained from mice receiving gefitinib showed a marked change in lipid expression compared to their predose profiles, and this became more pronounced with time, with lipid expression profiles not quite returning fully to their predose values by 24 h post drug administration.

**Figure 4:**
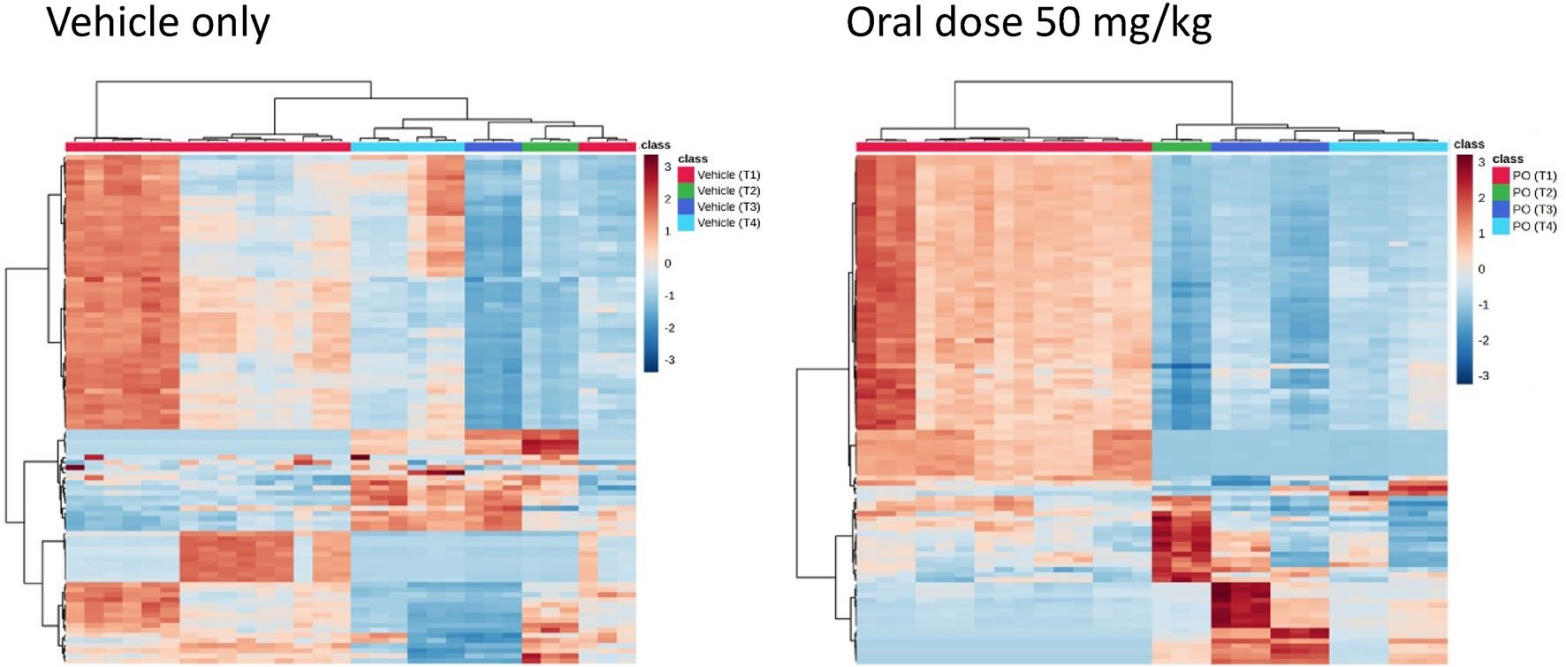
Heatmaps constructed using features consistent with the top 100 T-test/ANOVA values from the 50 mg/kg PO dose +ve ESI data. T1 = Predose, T2 = 3-h, T3 = 8-h and T4 = 24 h post dose. These heatmaps were produced using the top 100 t_R_–m/z 100 features identified by OPLS-DA as being responsible for differences between the plasma profiles of gefitinib dosed and control animals. These features are listed (for both +ve and −ve ESI) in the supplementary data (Table S4).

The +ve ESI data for the samples from the 50 mg/kg PO gefitinib-dosed mice also showed reductions in lipid abundances at the 3 h time point compared to their predose values, with lipid expression remaining unchanged at the 8 h time point and not returning completely to those obtained for predose samples by 24 h post dose (Fig. 4).

Using the +ve and −ve ESI data generated in these LC–MS studies the lipid category, class and function were identified by a combination of the exact mass, mass fragmentation pattern, chromatographic elution characteristics and database searches. The identity of each lipid was, where possible, confirmed by comparison of mass spectral data with those of authentic standards, following the guidelines outlined in^35^. Examples of the mass spectrometry data obtained from the analysis of lipids PC(40:2) (t_R_ = 3.27), PC(42:10) (t_R_ = 3.27), PC (38:6) (t_R_ = 1.97 min) and TG (56:8) (t_R_ = 4.55) are given in Figures S15–S18, (a worked example of the identification of the lipid feature eluting at t_R_ = 1.97 min is given in Figure S17). It is recognized that unequivocal identification of these lipids would require further isolation and analysis by targeted MS and NMR spectroscopy which was not possible with the limited amount of plasma available from this study.

### Pharmacometabodynamics

Following feature identification. for lipids subject to change following systemic exposure to gefitinib, it was apparent that representatives of a number of classes of lipids were affected. These included phosphatidylcholines (PC), phosphatidyl-ethanolamines (PE), phosphatidylinositol (PI), phosphatidylserines (PS) and triacyclglycerols (TGs) (see Tables 1, 2 and S1, S2). The responses of the affected lipids to the drug included increases or decreases in relative abundance, but with a general return towards predose values by 24 h post dose.

**Figure 5:**
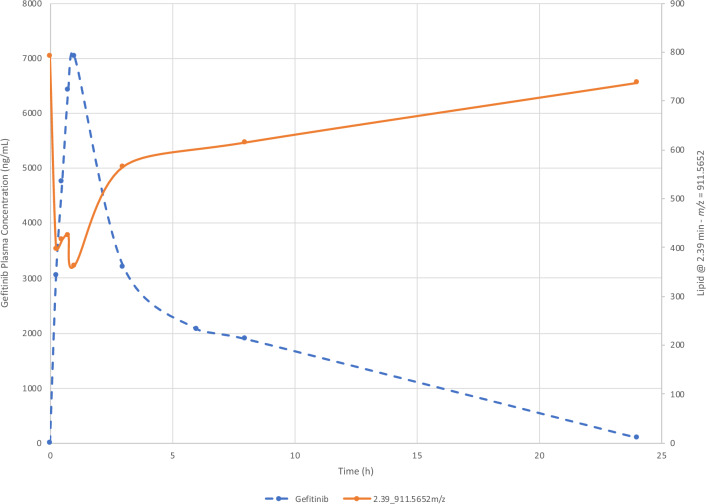
Change in response for lipid PI 40:5 following the oral administration of gefitinib (solid line). The pharmacokinetic profile for gefitinib (dotted line) following POadministration is included for reference.

**Figure 6:**
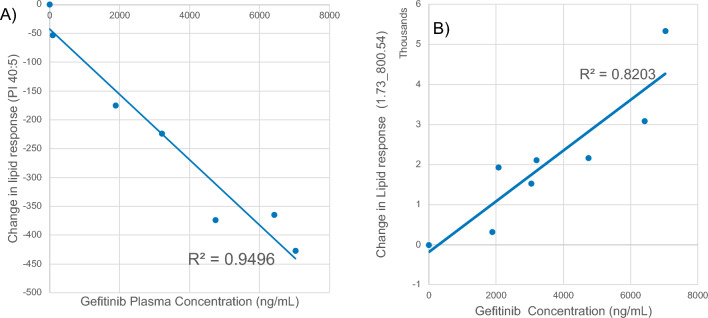
Relationship between the change in abundance for (**A**) PI (40:5) and (**B**) PC (34:3) with gefitinib concentration following the oral administration of gefitinib at 50 mg/kg to male mice.

Examples of the changes in normalized lipid response with time following the oral administration of gefitinib for lipids detected in −ve ESI, Table 2, are given in Figure S19 (for reference the pharmacokinetic profile of gefitinib for the PO dose is also included in this figure). Figure S19 was produced by the subtraction of the normalized predose lipid response for each lipid from the normalized lipid response obtained at the individual sampling time points. When compared to drug concentrations the time-related changes in response for some lipids, such as e.g., the t_R_–*m/z* pair 2.39_911.5652, detected using −ve ESI, corresponding to PI(40:5) (MS data shown in Figure S20), clearly shows an inverse response to the pharmacokinetic profile of gefitinib obtained after PO administration (Fig. 5). The relative abundance of PI(40:5) exhibited a rapid decline from the predose value, reaching an initial minimum abundance at the 0.25 h time point and a lowest abundance at the 1 h post dose. From this minimum PI(40:5) then increased rapidly in abundance to the 3 h post dose time point followed by a slow rise thereafter finally achieving a similar response to the predose value by 24 h after gefitinib administration (when the drug concentration was very low). This suggests that there was a direct causal link between the systemic concentrations of the drug and the changes in abundance of PI(40:5). When the changes in normalized lipid response (lipid response at t_X_–lipid predose response) for PI(40:5) were compared to the measured systemic concentrations of gefitinib, from predose to 24 h post dose, the results showed an inverse linear response to drug plasma concentration, with lipid abundance reducing as gefitinib concentration increased. The trendline for the PI(40:5) data showed a correlation coefficient of 0.9496 with and intercept of −42.35 (Fig. 6A). As the data points were evenly distributed above and below the trendline there appears to be no significant bias between the early and late time points as the systemic gefitinib concentration increased and declined.

Similarly the t_R__*m/z* pair 3.27_844.6042, attributed to PE(40:2) initially exhibited a marked drop in relative response following the PO administration of gefitnib, returning slowly to the predose value by 6 h, before falling again in abundance at 8 h post dose and remaining at that level at 24 h (Figure S21). PS(34:0) (t_R__*m/z* pair 1.91_808.5326) (Figure S22) also showed this pattern of an initial reduction in response to a minimum at 1 h post dose before increasing in intensity to 3 h, followed by a further decline in response in the 6 h sample (Figure S23). The decrease in relative abundances of these lipids at the 6 h time point mirrors the increases in concentration seen for gefitinib and the O-desmethyl metabolite shown in Figure S3 (which may have been due to enterohepatic recirculation of the drug and its metabolites^9^).

Whilst the changes in lipid abundance described above mostly showed relative declines in lipid abundance it was, in contrast, interesting to note that the feature detected at t_R_/*m*/*z* = 1.73_800.5430 showed show a positive time-related change in abundance which clearly tracked with the pharmacokinetic profile of gefitinib after oral administration (Figure S19). The relative abundance of this feature (1.73_800.5430) exhibited a steady increase from the predose value reaching a maximum abundance at 1 h post dose point before declining thereafter (see Figure S19). Examination of the mass spectral data for this lipid indicated that it was PC(34:3) (Figure S24). When the changes in the normalized signal response for this lipid were compared to the measured systemic concentration of gefitinib from predose to 8 h post dose the data trendline showed a correlation coefficient of 0.8203 with and intercept of −187.58 (Fig. 6b).

These results showing an apparent correlation, either positive or negative, with plasma lipid abundances and drug concentrations are similar to those observed for more polar metabolites such as tryptophan and taurocholic acid excreted in the urine samples from this study^19^. These lipidomic data thus appear to confirm the direct link between the pharmacokinetics of gefitinib and the changes in the lipid abundance. This phenomenon, which we have previously referred to as pharmacometabodynamics when discussing more polar metabolites, would in the case of lipids, perhaps be better characterized here by the term “pharmacolipidodynamics".

## Discussion

Metabonomics/metabolomics, lipidomics and proteomics are increasingly recognized as providing powerful tools for the investigation of drugs and their effects on mammalian biology^20–22^. These Omics methods now have many applications in assessing disease progression, population health, gender/ethnicity-based variations in response, and treatment effectiveness etc.,^23–27^. In their review of the impact of TKI’s on metabolic pathways in cancer Poliaková et al.,^18^ noted that “therapeutic responses to TKI’s are primarily linked to such pathways as regulation of lipid and amino acid metabolism, TCA cycle and glycolysis”. Indeed, cancer tumour progression is well known to affect lipid synthesis e.g.,^28^ and lipid profiling has been used to identify biomarkers in cancer^29–32^. However, as noted by Poliaková et al., “our current expertise regarding alterations in lipid metabolism upon distribution of distinct TKIs is rather limited”^18^. Here, in non-tumour-bearing mice, we have demonstrated using multivariate statistical analysis of the LC–MS data acquired from gefitinib-dosed animals that gefitinib had a range of effects on several lipid classes following the PO and IV administration of the drug to male C57Bl/6JRj mice. The affected lipids included representatives of the PI, PC, LPC, PE and TG, which collectively participate in a wide range of cellular activities including the formation, structure and function of cell membranes. Glycerophospholipids are the major lipid component of cell membranes^33^ whilst the PI play a key role in lipid and cell signalling as well as membrane trafficking etc.,^34,35^ and the PCs play an important role in membrane-mediated cell signalling and the activation of enzymes^36^. Others, such as e.g., the LPC have been linked to the activation of endothelial cells during early atherosclerosis^37^ whilst the TG provide a rich source of energy but are also associated with many disease states such as atherosclerosis etc.,^38,39^. In this study there appeared to be an inverse relationship between the abundance of PI(40:5) and systemic gefitinib concentration following oral administration suggesting a causal effect. Phosphoinositides, such as PI(40:5) are found in cell membranes, where they have an important role in lipid-protein interactions. Llorente et al.^40^ reported that KRAS targeting compounds have been shown to reduce the concentration of PIs in cancer tumours. Furthermore, Uribe et al., reported that EGFR inhibitors, such as gefitinib, have been shown to reduce levels of phosphatidylinositol (3)-monophosphate in tumours^41^ thus the observed reduction in PI abundance following gefitinib administration to mice is in line with these previous observations.

Overall, the widespread effects of gefitinib on phospholipids seems, on the basis of the data obtained here, to have been a reduction in the amount of the affected lipids compared to vehicle-dosed mice. The exception to this pattern of reduced plasma abundance of lipids in the circulation seems to be with respect to the PEs where a number (but not all) of this class that were identified as being affected by drug administration increased in the plasma of gefitinib dosed animals (see Tables 1 and 2). The reasons for, and the consequences of, these increases in PEs such as PE (40:6) and PE (41:6), which showed changes of between 1.2 and 1.4 fold for the 1–3 h post dose time points following PO administration (Table 1), are a matter of conjecture. However, the demonstration of these effects of gefitinib on lipid metabolism represent an intriguing insight into the potential mode(s) of action of this TKI inhibitor with relation to role of EGFR in cancer. Gottschalk et al.reported that exposure of the TKI inhibitor imatinib to BCR-ABL-positive cells resulted in a significant upregulation of GPC and a decrease in PC abundances^27^. Such an effect is potentially important as PC’s have been shown to accumulate in breast and colon tumours^42^, possibly as a result of enhanced choline transport. Similarly, Zheng et al.^42^ have shown that the treatment of hepatocellular carcinoma cells (HepG2) with the multikinase inhibitor sorafenib affected glycerophospholipid metabolism. In contrast non tyrosine kinase inhibitors, targeted to e.g., PI3K and RAS resulted in a reduction in choline-containing lipids, comprising total choline, phosphocholine and glycerophosphocholine^43^.

Whilst the changes in the plasma lipidome resulting from gefitinib administration revealed in the present study do indicate that the drug has effects on lipid metabolism they cannot be claimed to fully address the limitations highlighted by Poliaková et al., in our knowledge with respect to TKI inhibitors^18^. A limitation of the present work is that the small amount of sample remaining after the bioanalysis and drug metabolism studies were completed (9) precluded a more in-depth study of the effects of gefitinib on the plasma lipidome. However, these preliminary results provide support for further in vitro and in vivo studies (including cell lines and tumor models), on gefitinib and a range of similar inhibitors that would clearly be helpful in providing a further understanding of the metabolic effects of this class of drug. In particular, having identified classes of lipid where changes in abundance had occurred, a more targeted and quantitative study, that resulted in firm lipid identifications, would be beneficial.

There is also clearly a need, given the differences in dose and exposure to gefitinib between experimental animals and humans, where plasma concentrations of the drug are lower than those seen here (e.g., see^44^), to see if similar effects on the lipidome are observed in patients.

The potential of untargeted metabolic and lipid profiling to investigate and predict the pharmacological effects of pharmaceuticals (through e.g., pharmacometabonomics^46^) has long been evident. The pharmacolipidodynamic effects revealed in this study, directly linking circulating drug concentrations to effects on plasma lipids adds a further dimension to this type of work. Given the impressive sensitivity of current analytical methods, which require only a few microlitres of plasma, such analyses can be undertaken without increasing animal use, by re-analysing the samples obtained for other purposes in e.g., preclinical pharmacology drug metabolism studies. The same methods can later be applied to samples obtained from for clinical studies to ensure that the results obtained in drug discovery translate to humans^45^.

## Conclusion

The TKI inhibitor gefitinib administered, by either the IV or PO, routes to male C57Bl/6JRj mice resulted in significant changes to the plasma lipid profiles obtained by LC–MS. Multivariate statistical analysis of the resulting lipidomic data, for both routes of administration, showed a time-related trajectory with major effects association with the high drug concentrations attained soon after drug administration. By 24 h post dose the effects of gefitinib on the 10 mg/kg IV plasma lipid profile had generally dissipated and it had largely returned to its predose condition. In the case of the 50 mg/kg, PO dose similar changes in lipid profiles were seen but a return to the predose condition was not obtained by 24 h post dose. Investigation of the lipids responsible for the variance revealed that the PE, PI, PC and LPC profiles were the most disrupted by the administration of this TKI inhibitor, indicating a strong link between EGFR and lipid metabolism. The correlation of the pharmacokinetic profile of gefitinib with the observed plasma lipid changes provides further evidence for a pharmacometabodynamic link.

## Materials and methods

LC–MS grade water, isopropanol (IPA), methanol (MeOH), acetonitrile (ACN), ammonium acetate, formic acid (FA) and leucine enkephalin (LeuEnk) were sourced from Thermo Fisher Scientific (Franklin, MA, USA). Sodium formate, used to calibrate the time-of-flight (TOF) mass spectrometer, was sourced from Waters Corporation (Milford, MA, USA). Distilled water was obtained via an in-house Millipore System (Millipore, Burlington, MA, USA). SPLASH®, LIPIDOMIX® Mass Spec Standard (cat # 330707) was obtained from Avanti® Polar Lipids (Birmingham, AL, USA).

### Mouse plasma samples

Mouse plasma samples were obtained in a previously reported metabolism pharmacokinetic study of gefitinib in 30 male C57Bl/6JRj mice (20–27 g) in 3 groups (10 in each of the vehicle, IV and PO groups) (a full description of the study can be found in^9,19^). For PO administration (50 mg/kg) of a homogeneous suspension in hydroxypropyl-methylcellulose was used. For IV dosing (10 mg/kg) a solution in aqueous hydroxypropyl beta-cyclodextrin and acetate buffer (pH 4.0 50 mM) 10:90 w/v was used. The mice were housed, by dose route, in groups of 5 in glass metabowls. Blood was sampled (50 µL) via the tail vain on two occasions per animal during the course of the study with 100 µL taken at termination. Blood samples were taken pre-dose, (0.1; IV only), 0.25, 0.50, 0.75, 1, 2, 3, (6; PO only), 8, and 24 h post-dose (2 mice/time point, 1 mouse/cage) for control, PO an IV routes of administration. The plasma samples were prepared by centrifugation at 2500*g* and stored frozen at −80 °C prior to analysis. The study (DMPK-2019-669) was performed with full management and ethical review and according to national and EU guidelines by Evotec SAS (Toulouse, France). The animal experimental protocols were approved by Evotec SAS. The study is reported in accordance with the ARRIVE guidelines 2.0, (https://arriveguidelines.org/arrive-guidelines).

### Plasma sample preparation

Mouse plasma samples were prepared by mixing 20 µL of sample with 100 µL of IPA at 4 °C to precipitate the plasma proteins. When the amount of sample available was less than 20 µL the samples were mixed with an appropriate volume of IPA to maintain a sample:solvent volume ratio of 1:5 (v/v). The samples were vortex mixed for 60 s and transferred to the freezer at −20 °C for 10 min, after which they were vortex mixed again and placed in the refrigerator for 2 h at 4 °C to effect complete precipitation of the plasma proteins. The samples were then centrifuged at 10,300*g* for 10 min, at 4 °C, and the supernatant layer removed, this was then diluted 1:5 (v/v) with IPA:ACN (1:1 v/v). The resulting samples were then transferred to Total Recovery UPLC Vials (Waters Corp, Milford, USA) for analysis. The samples were randomized for analysis with each sample being analysed in triplicate. A pooled QC, used to monitor the performance of the analysis, was prepared by mixing 10 µL of plasma from each mouse at each time point, this sample was prepared in the same manner as the other study samples^46^.

### System suitability testing

Prior to analysis the SPLASH LIPIDOMIX (composed of deuterated lipids) was employed as a systems suitability test (SST) using a solution containing between 250 and 1000 ng/mL, depending upon the lipid, in IPA:ACN (1:1 v/v). The SST was used to confirm that mass accuracy, retention time (t_R_) and LC peak shape/intensity were acceptable. An example of the chromatographic profile obtained for selected lipids in the SPASH MIX is shown in Figure S25. The SPLASH LIPIDOMIX was also added, at the same concentration as the SST to both the pooled QC (Figure S26) and individual plasma extracts (Figure S27)  for potential use as a tool for rapidly detecting and investigating unexpected changes in instrument performance that had occurred during sample analysis.

### Sample analysis

Sample analysis was performed by UHPLC-MS as described below. Prior to beginning the analysis of the samples two “solvent blank” extractions, consisting of the starting composition of the mobile phase were made, followed by 2 injections of an “extraction” blank, 2 injections of the SST and finally 4 injections of the pooled QC sample. The randomized study samples were then analysed (in triplicate) including a pooled QC every 10th study sample. On completion of the analysis of the study samples a further 5 pooled QC samples were analysed followed by two solvent blanks, 2 extraction blanks and finally a single injection of the SST. Typical mass chromatograms for the SST used as such and in the QC and individual animal samples obtained during this analysis are shown in Figures S25, S26 and S27 respectively for +ve ESI, a summary of the variation in the detector response from selected lipids from the SPLASH LIPIDOMIX is given in Table S3.

### Chromatography

The chromatographic analysis was performed on a Waters ACQUITY™ UPLC™ Premier system equipped with a flow through needle (Waters Corp, Milford, USA). The separations were performed using a vacuum jacketed (see ref^47^) stainless-steel 2.1 × 50 mm ACQUITY CSH™ C_18_ 1.7 µm column (Waters Corp, Milford, USA). The vacuum jacketed column was located on the source of the mass spectrometer with the column effluent being directly transferred to the MS probe via a short length of capillary tubing, (6 cm of 50 µm ID tubing from the column to the probe followed by a 35 cm length of 75 µm ID within the probe). The vacuum jacketed column inlet temperature was 70 °C with an outlet temperature of 80 °C.

The mobile phases used for chromatography comprised ACN:H_2_O:1.0 M aqueous ammonium formate in the ratio 600:390:10; v/v/v containing 0.1% FA (mobile phase A) and IPA/ACN/1.0 M aqueous ammonium formate (900:90:10; v/v/v) containing 0.1% FA (mobile phase B) giving a final concentration of ammonium formate of 10.0 mM in both. The solvent was delivered at a flow rate of 0.5 mL/min, following a 2 µL injection of the sample and eluted using the multi-linear gradient detailed in Table 3, whilst the MS data were collected for 5 min. This methodology was adapted from that previously described^48^.

**Table 3:** Chromatographic conditions.

Time (min)	Flow (mL/min)	%A	%B
Initial	0.5	50	50
0.25	0.5	47	53
2	0.5	45	55
3.5	0.5	35	65
3.75	0.5	20	80
5	0.5	1	99
6	0.5	1	99

### Mass spectrometry

Mass spectrometric analysis was performed on a Xevo™ G2-XS QTof (Waters Corporation, Wilmslow, UK) using both +ve ESI and −ve ESI. Capillary voltages of 3.0 kV for +ve ESI and 2.5 kV for −ve ESI were employed. The source temperature was maintained at 100 °C and a cone gas (N_2_) flow of 50 L/h was used. The desolvation gas (N_2_) was operated at 600 L/h with a temperature of 300 °C, whilst the desolvation and nebuliser gases were set at 6 bar. MS data were acquired over the m/z range 50–1200 Da for both modes of ionization. Sodium formate was employed to calibrate the TOF detector. Data were collected in continuum mode using alternate high/low collision energy switching (MS^E^). The low collision energy was set to 4 eV (function 1) with a collision energy ramp (25 to 45 eV) used for the elevated energy data (function 2). A scan time of 0.1 s was employed for both functions, allowing for an optimal compromise between number of points acquired across chromatographic peaks and the ion statistics required for mass accuracy. LeuEnk (*m/z* 556.2771 (ESI+); *m/z* 554.2615 (ESI−)) was used as the external lock mass with a scan collected every 30 s at a cone voltage of 40 V.

### Data analysis

The data were collected using MassLynx™ vs. 4.1 (Waters Corp., Wilmslow, UK) whilst data processing and visualization was predominantly conducted using Progenesis™ QI vs. 3.0 (Nonlinear Dynamics, Newcastle-upon-Tyne, UK). The LC–MS data were chromatographically aligned (based on the pooled study QC), peak picked and normalised against all compounds using Progenesis QI (https://www.nonlinear.com/progenesis/qi/v1.0)^49^ prior to multivariate statistical analysis (MVA) and database searching. LipoStar (Molecular Discovery, Hertfordshire, UK) was also used for additional data processing and lipid identification. Statistical analyses of these data was undertaken using multivariate analysis performed with EZInfo version 2.0 (Sartorius, Gottingen, Germany) with the signals corresponding to gefitinib and its known metabolites (which eluted between 0.0–0.5 min) removed from the data before processing. The LC–MS raw data acquired for the pooled QC samples were then subjected to peak selection to define the peak picking parameters and the resulting mass retention time pairs (*m/z*–t_R_). The pooled QC sample was also used to determine features which had a CV > 20%, features above this threshold were eliminated from the subsequent data analysis. Principal component analysis (PCA) was performed using Pareto scaling over the data range of 0.5 – 6 min. Orthogonal projection to latent structure discriminant analysis (OPLS-DA) was also performed on selected data as indicated in the text using EZInfo software. Putative lipid identification was performed using a combination of online databases searches (Lipidblast, UC Davis, Davis, CA), Lipid Maps (Lipidomics Gateway, https://www.lipidmaps.org)^50^ and Chemspider (http://www.chemspider.com/Chemical-Structure.1906.html) using a precursor tolerance of 10 ppm and product ion tolerance of 15 ppm. Examination of the database suggestions was then undertaken using the mass spectral data (accurate mass of their precursor ions, fragment ion match and isotopic pattern) of individual compounds. Additional data interrogation and visualisation was performed using MetaboAnalyst (https://www.metaboanalyst.ca/)^51^. The data for the top 100 t_R_–m/z 100 features identified by OPLS-DA in both +ve and −ve ESI (listed in Table S1 of the supplementary data) responsible for differences between the gefitinib dosed and control mice was used for the construction of heatmaps to aid visualization (Figs. 3 and 4).

### Supplementary Information

Supplementary Information.

## Data Availability

The datasets generated and/or analysed during the current study are available in the EMBL-EBI, Wellcome Genome Campus, Cambridgeshire, UK repository, dataset identifier MTBLS8663, and are available upon request from rob_plumb@waters.com.
