# Supplementary material for: Detection of pharmacolipidodynamic effects following the intravenous and oral administration of gefitinib to C57Bl/6JRj mice by rapid UHPLC-MS analysis of plasma
Source: Sci Rep. 2024 Jul 24;14:17061. doi: 10.1038/s41598-024-66764-w (PMC11269747; doi:10.1038/s41598-024-66764-w)
Supplement: Supplementary file 1 — Supplementary Information. [file 41598_2024_66764_MOESM1_ESM.docx]

**Supplementary Information**

**Detection of Pharmacolipidodynamic Effects Following the Intravenous and Oral Administration of Gefitinib to C57Bl/6JRj Mice by Rapid UHPLC/MS Analysis of Plasma**

Robert S Plumb^1^, Lee A Gethings^2^, Giorgis Isaac^3^ Nyasha C Munjoma^2^, Ian D Wilson^4^

1. Waters Corporation, Milford, MA, 01757, USA
2. Waters Corporation, Stamford Ave, Wilmslow, SK9 4AX, UK
3. Program in Molecular Medicine, University of Massachusetts, Chan Medical School, 373 Plantation Street, Worcester, MA 01605, USA
4. Computational & Systems Medicine, Department of Metabolism, Digestion and Reproduction, Imperial College, Burlington Danes Building, Du Cane Road, London, W12 0NN, UK

*Corresponding Author i.wilson@imperial.ac.uk

Figure S1: Structure of Gefitinib


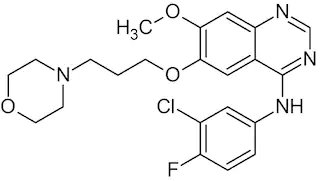


Figure S2: Pharmacokinetics of Gefitinib following IV administration at 10mg/kg to male C57Bl/6JRj mice

Figure S3: Pharmacokinetics of Gefitinib following PO administration at 50mg/kg to male C57Bl/6JRj mice

Figure S4: Positive ion LC-MS ESI chromatogram of pooled plasma QC sample formed from male C57Bl6 mice dosed with gefitinib 10mg/Kg (IV), 50mg/Kg (PO) and vehicle only.

Triacylglycerols

Cholesterol esters

Lysophospholipids

Phospholipids

Sphingomyelins

Figure S5: Negative ion LC-MS ESI chromatogram of pooled plasma QC sample formed from male C57Bl6 mice dosed with gefitinib 10mg/Kg (IV), 50mg/Kg (PO) and vehicle only.

Lysophospholipids

Free Fatty Acids

Sphingomyelins

Phospholipids

Figure S6: PCA of +ve ion LC-MS ESI data for plasma obtained from male C57Bl6 mice dosed with vehicle for the periods of predose, 0.25, 0.5, 0.75, 1, 2, 3, 6, 8 and 24 h.  These data were obtained following triplicate analysis of plasma samples.  Animal cages are identified using the notation (A) and (B).

Note that in this, and Figures S7-10, the coloured ellipses have no statistical significance but are an aid to identifying the results for samples from the same time points obtained pre- and post-vehicle or gefitinib administration.

Figure S7: PCA of +ve ion LC-MS ESI data for plasma obtained from male C57Bl6 mice dosed with vehicle for the periods of predose, 0.25, 0.5, 0.75, 1, 2, 3, 6, 8 and 24 h. These data were obtained following triplicate analysis of plasma samples. Animal cages are identified using the notation (A) and (B).

Figure S8: PCA of +ve ion LC-MS ESI data for plasma obtained from male C57Bl6 mice following the IV administration of gefitinib at 10mg/kg for the periods of predose, 0.25, 0.5, 0.75, 1, 2, 3, 6, 8 and 24 h. These data were obtained following triplicate analysis of plasma samples. Animal cages are identified using the notation (A) and (B). The results show a clear time related trajectory in the data, from predose to 6 h with the 8 and 24 h samples returning close to the predose position.

Figure S9: PCA of -ve ion LC-MS ESI data for plasma obtained from male C57Bl6 mice following the oral administration of gefitinib at 50mg/kg for the periods of predose, 0.25, 0.5, 0.75, 1, 2, 3, 6, 8 and 24 h. These data were obtained following triplicate analysis of plasma samples. Animal cages are identified using the notation (A) and (B). The data shows the cage effects and a time related trajectory in the data following dosing, note that after 24 h the samples have not returned to their predose levels.

Figure S10: A) PCA of +ve LC-MS ESI data for plasma obtained from male C57Bl6 mice following the oral administration of gefitinib at 50mg/kg for the periods of predose, 0.25, 0.5, 0.75, 1, 2, 3, 6, 8 and 24 h. These data were obtained following triplicate analysis of plasma samples. Animal cages are identified using the notation (A) and (B). These data show the cage effects and clear presence of a time related trajectory in the data following dosing.

Figure S11: PCA (PC1 vs PC2 vs PC3) of the +ve ion LC-MS ESI data for plasma obtained from male C57Bl6 mice following the IV administration of gefitinib at 10mg/kg for the periods of predose, 0.25, 0.5, 0.75, 1, 2, 3, 6, 8 and 24 h. The data illustrate that after 24 h (pale blue circles) the lipid chemistry of the mice has not returned to it predose (black circles) values.


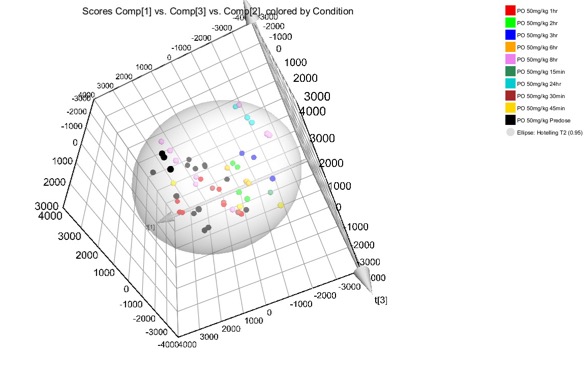


Figure S12: A) OPLS DA of -ve LC-MS ESI data for plasma obtained from male C57Bl6 mice following the IV administration of gefitinib at 10mg/kg for the periods of predose vs 0.1 + 0.25 h. These data were obtained following triplicate analysis of plasma samples. The areas enclosed by the rectangles show ion features that showed the greatest variance both statically and in terms of relative abundance for the 0.1 and 0.25 h samples (red rectangle) and predose samples (blue rectangle). B) Analysis of the same data but without removal of the gefitinib-related ions.

A)

B) Gefitinb-related ions

Figure S13: OPLS DA of the -ve ion LC-MS ESI data for plasma obtained from male C57Bl6 mice following the IV administration of gefitinib at 10mg/kg for the periods of predose vs 1 + 3 h time points. These data were obtained following triplicate analysis of plasma samples. The areas enclosed by the rectangles show ion features that showed the greatest variance both statically and in terms of relative abundance for the 1 and 3 h samples (red rectangle) and predose samples (blue rectangle).

Figure S14: OPLS DA of the -ve ion LC-MS ESI data for plasma obtained from male C57Bl6 mice following the oral administration of gefitinib at 50mg/Kg for the periods of predose vs 3 h time points. These data were obtained following triplicate analysis of plasma samples. The areas enclosed by the rectangles show ion features that showed the greatest variance both statically and in terms of relative abundance for the 1 and 3 h samples (red rectangle) and predose samples (blue rectangle).

Figure S15: MS2 spectrum obtained from the peak eluting at t_R_ = 3.27 min from the -ve ion LC-MS ESI analysis of plasma from C57Bl6 mice following the oral administration gefitinib at 50mg/kg. The MS/MS spectra obtained for PC (40:2) was compared to that of an authentic standard in Lipostar database.


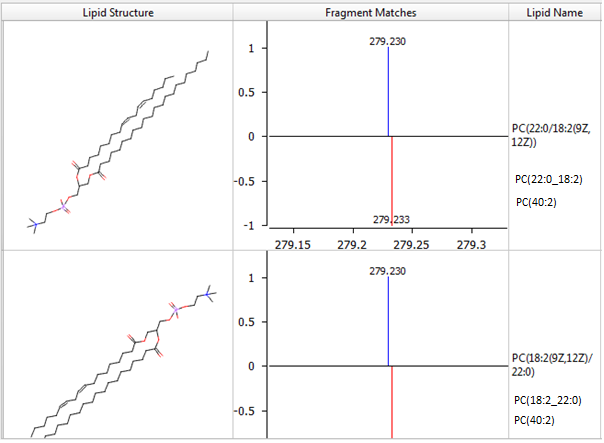


*This identification is provided directly from LipoStar and the correct lipid name is denoted as PC(18:2_22:0). The fragment ion at m/z 279.230 corresponds to the RCOO-loss from the sn1 chain.

Figure S16: MS2 spectra obtained from the peak eluting at t_R_ = 3.27 min from the -ve ion LC-MS ESI analysis of plasma from C57Bl6 mice following the oral administration gefitinib at 50mg/kg. The MS/MS spectra obtained for PC(42: 10) was compared to that of an authentic standard in the Lipostar database.


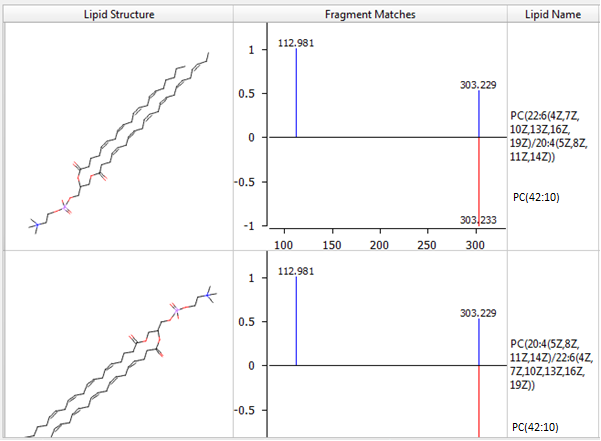


This identification was provided directly from LipoStar and the correct lipid name is denoted as PC(20:4_22:6). The fragment ion at m/z 303.229 corresponds to the RCOO-loss from the sn1 chain.

Figure S17: MS2 Spectrum obtained from the peak eluting at t_R_ = 1.97 min from the +ve ESI analysis of plasma from C57Bl6 mice following the oral administration of gefitinib at 50mg/kg. The Progenesis QI MS/MS spectra obtained for PC(38:6) is shown below.


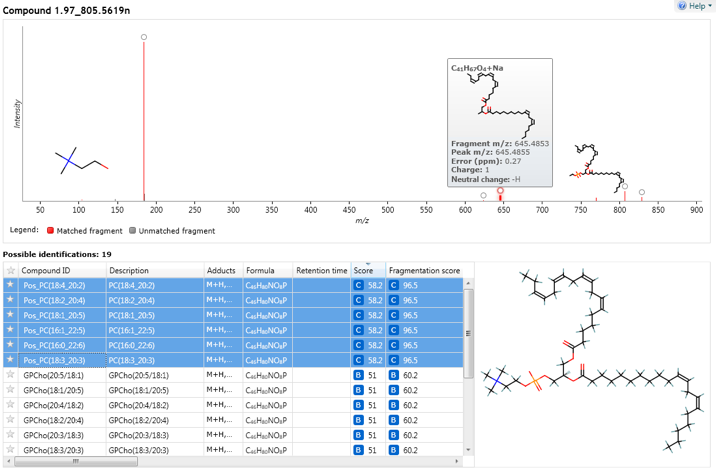


Analysis of this feature showed that this lipid had a precursor neutral mass of 805.5619, giving an elemental composition of C46H80NO8P, with a mass error of 0.3 mDa. Two major fragment ions were obtained for this lipid, one at m/z = 184.0740 which was attributed to the loss of the choline headgroup and the other, of m/z = 645.4853, which was considered to be the co-eluting sodium adduct of C41H67O4 with a mass error of 0.27 ppm. The top six database lipid identifications for this feature returned the same result, a PC (38:6) with various different combinations for the position of unsaturation.

Figure S18: MS2 Spectrum obtained from the peak eluting at t_R_ = 4.55 min from the +ve LC-MS ESI analysis of

plasma from C57Bl6 mice following the oral administration gefitinib at 50mg/kg.

The Progenesis QI MS/MS spectrum obtained for TG(56: 8) is shown below.


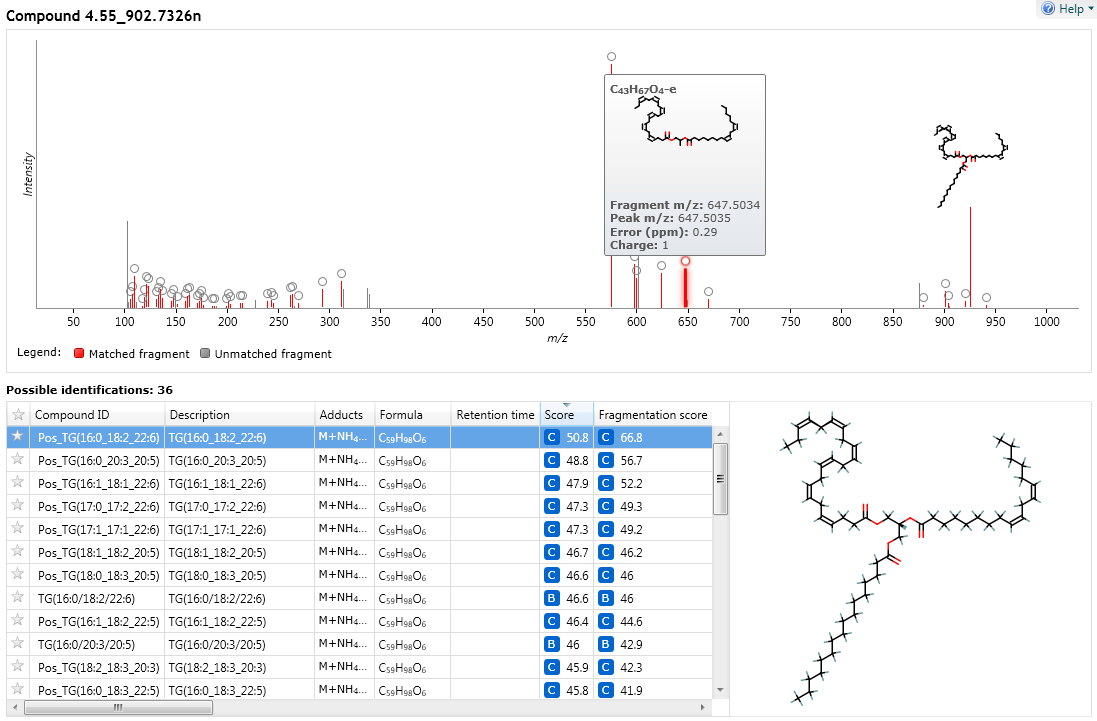


The precursor ion observed at *m/z* 920.7326 (as the ammoniated adduct) showed dominant fragment ions correlating to the TG(56:8) identifications (above) show diagnostic fragment ions at *m/z* 647.5, 623.5 and 575.5 correspond to the neutral loss of RCOOH+NH_3_ from the sn1→sn3 chains. Many less abundant ions, below *m/z* 400, are RC=O losses from acyl chains. Further data from the Lipostar database below


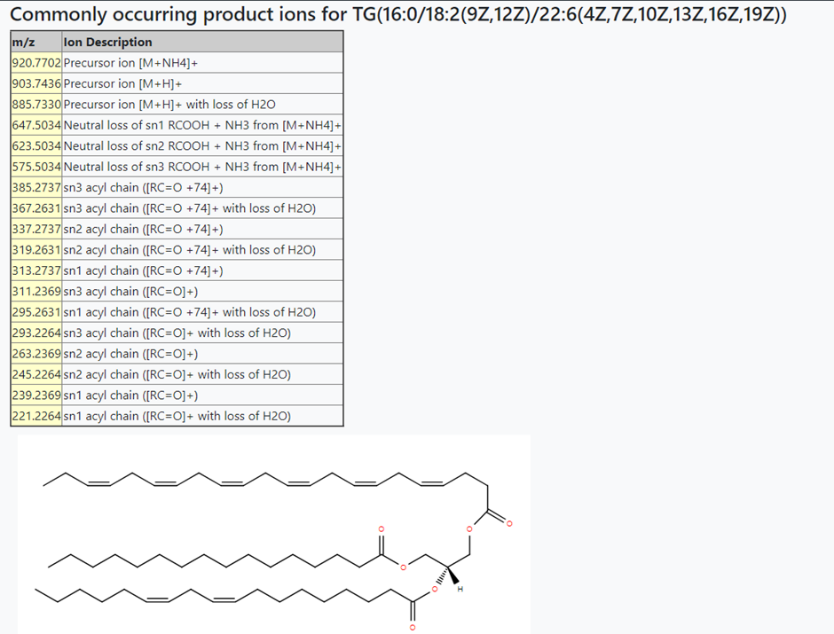


Figure S19: Change in lipid abundances and gefitinib concentrations following the oral administration of gefitinib at 50 mg/kg.

Figure S20: MS2 Spectrum obtained from the peak eluting at t_R_ 2.39 min from the -ve

ion LC-MS ESI analysis of plasma from C57Bl6 mice following the oral

administration gefitinib at 50mg/kg. The MS/MS spectra obtained for PI(40:5)

was compared to that of an authentic standard in Lipostar database.


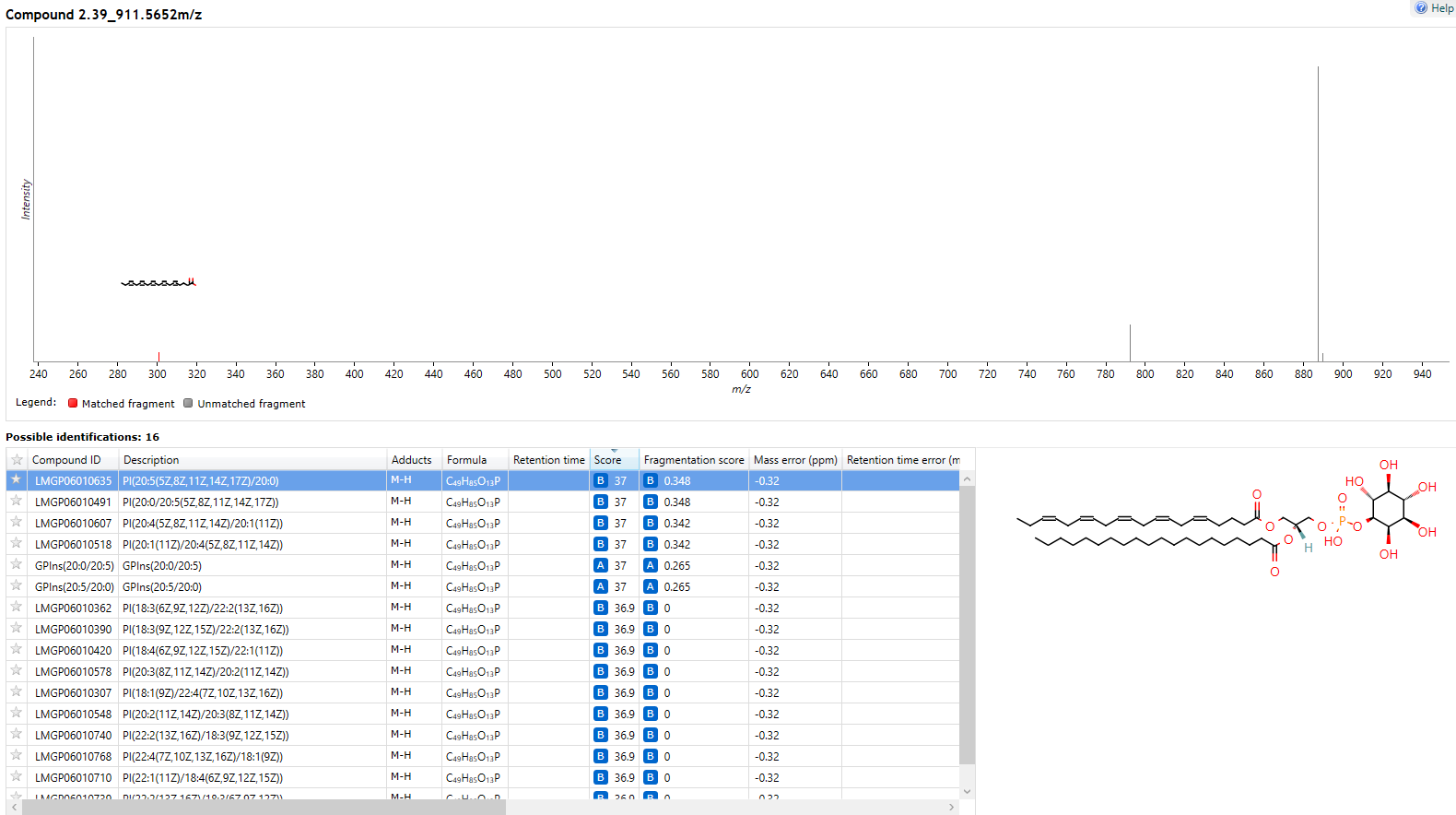


Figure S21: Change in response for lipid PE 40:2 following the PO administration of

gefitinib at 50 mg/kg (pharmacokinetic profile shown for

reference).

Figure S22: MS2 Spectrum obtained from the peak eluting at t_R_ 1.91 min from the -ve

ion LC-MS ESI analysis of plasma from C57Bl6 mice following the oral

administration gefitinib at 50mg/kg. The MS/MS spectra obtained for PS(34:0)

was compared to that of an authentic standard in Lipostar database.


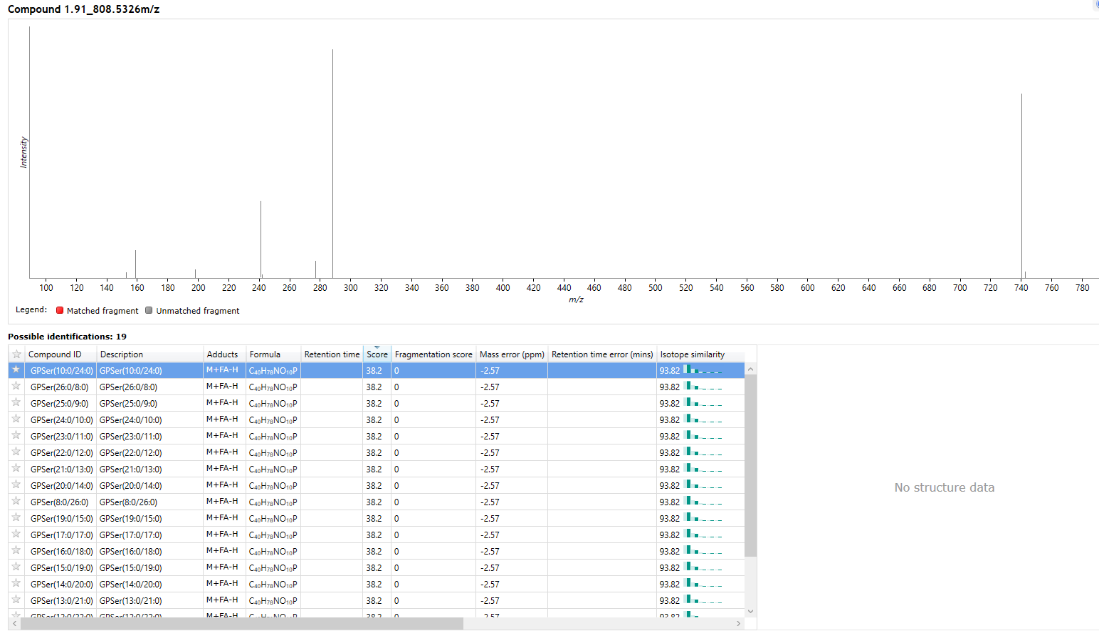


Figure S23: Change in response for lipid PE(34:0) following the PO administration of gefitinib at 50 mg/kg (pharmacokinetic profile of gefitinib included for reference).

Figure S24: MS2 Spectrum obtained from the peak eluting at t_R_ 1.73 min from the -ve

ion LC-MS ESI analysis of plasma from C57Bl6 mice following the oral

administration gefitinib at 50mg/kg. The MS/MS spectra obtained for PC(34:3)

was compared to that of an authentic standard in Lipostar database.


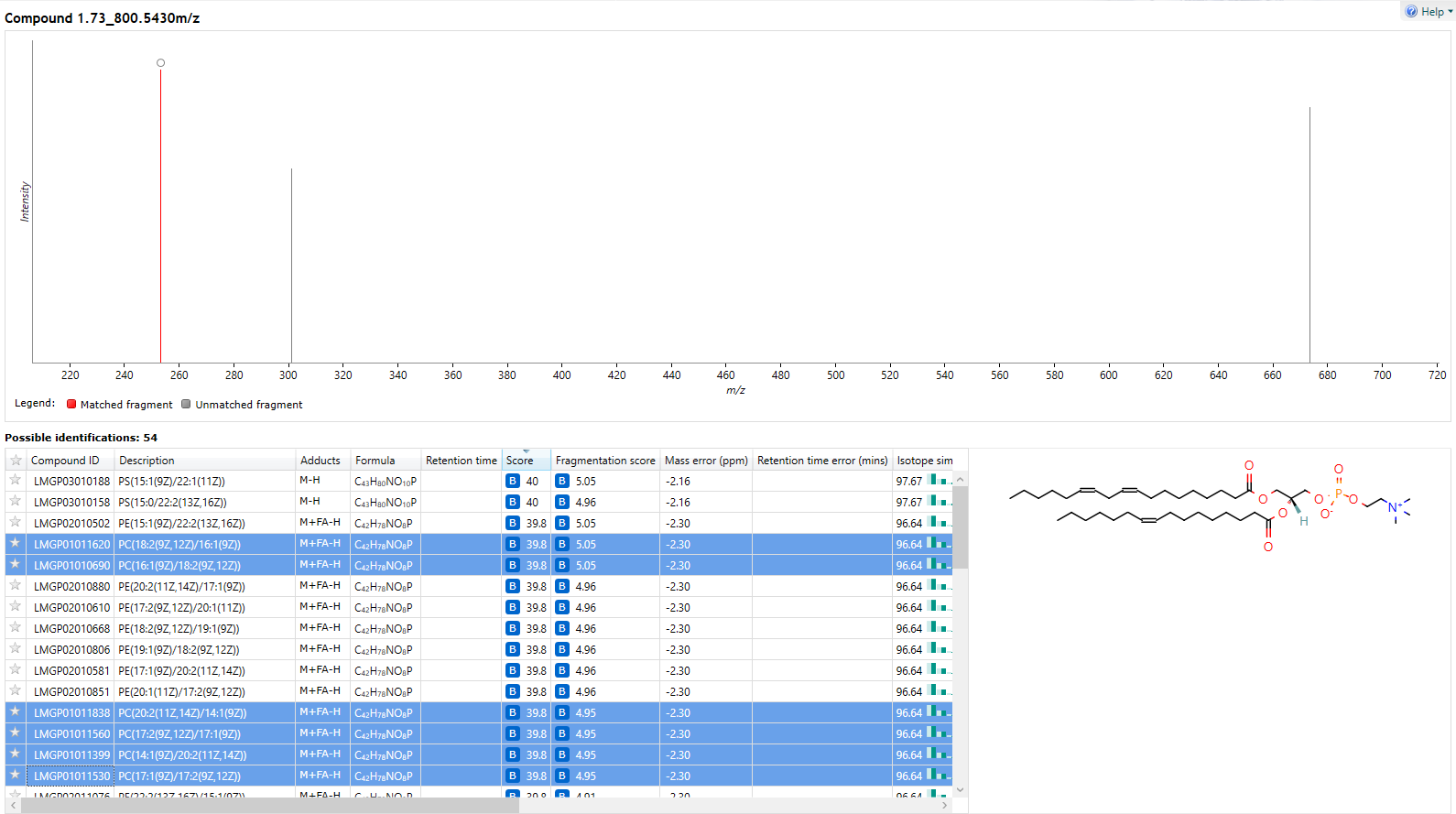


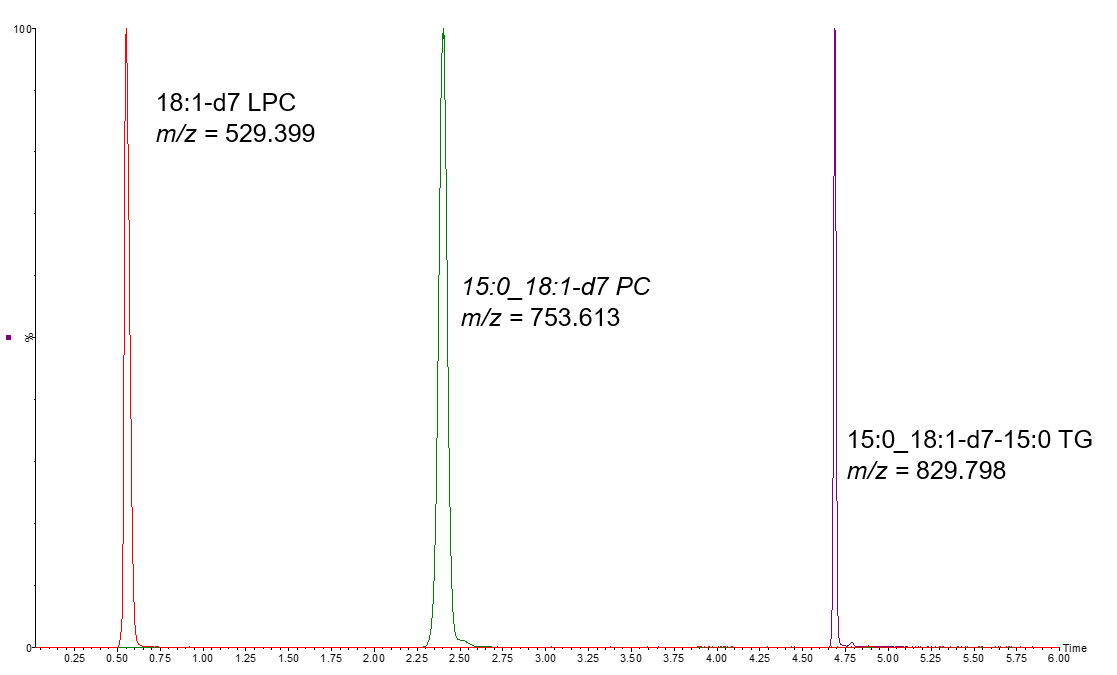


Figure S25: Mass chromatogram showing the results for 3 of the deuterated lipids analysed in the SST under the chromatographic conditions employed for the analysis of mouse plasma. The SPLASH LIPIDOMIX was dissolved in IPA:ACN (1:1 v:v)


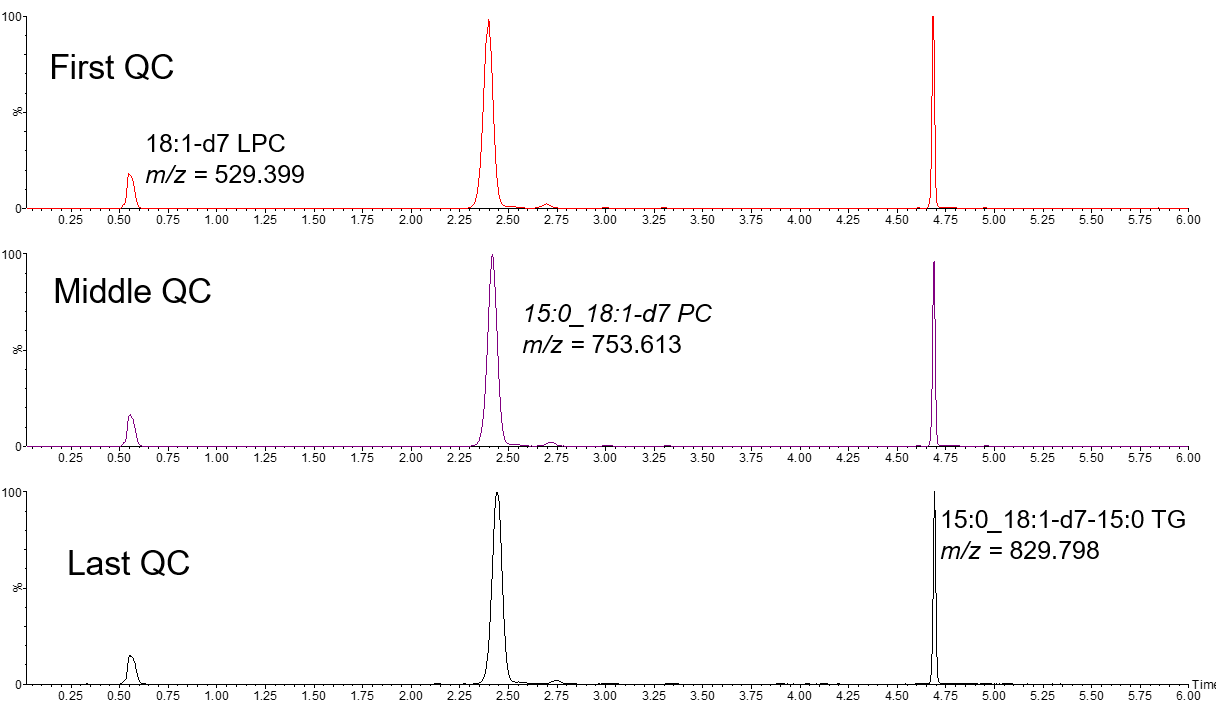


Figure S26: Mass chromatograms for 3 of the deuterated lipids covering time course of the UHPLC-MS analysis of mouse plasma for QC samples spiked with the SST. These results show that t_R_, peak shape and signal intensity were unchanged during the course of the analysis. Note the reduced intensity of the LPC 18:1-d7 in the plasma extract compared to the SPLASH LIPIDOMIX in IPA:ACN (1:1 v:v) which we attribute to matrix-related effects (ion suppression) due to the elution of the lipid near the solvent front.


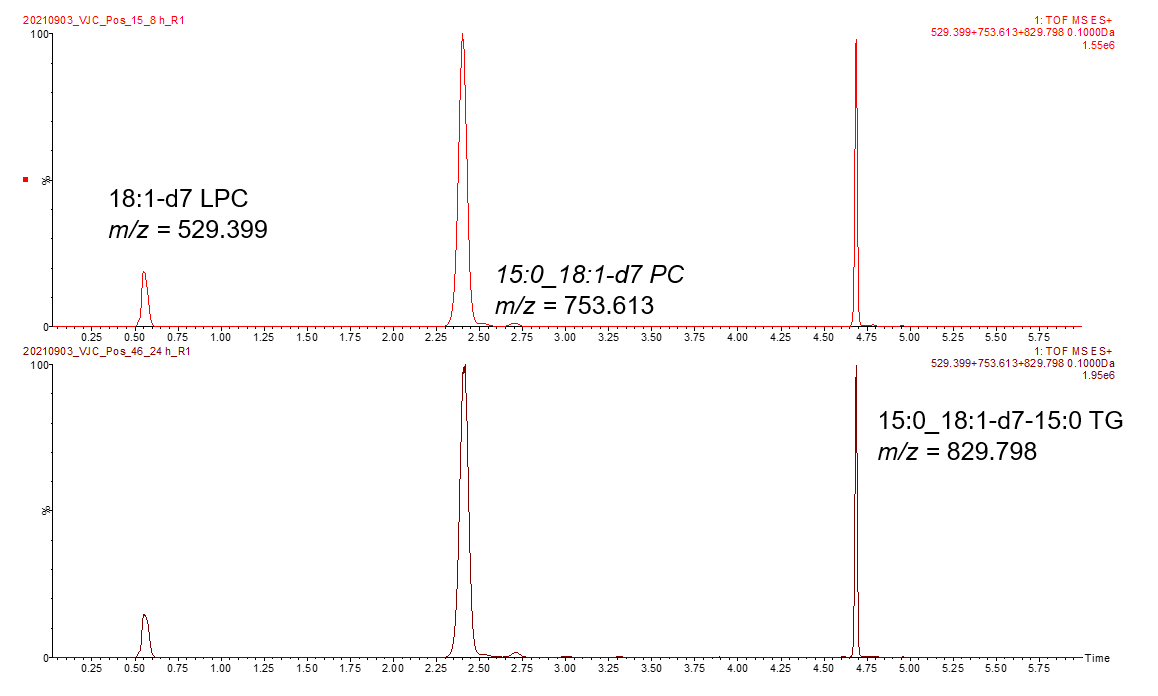


Figure S27: Mass chromatogram showing the results for the deuterated lipids highlighted in Figure S25 obtained for two plasma mouse samples analysed in the study. These results provide further evidence to complement those of the QC samples for the stability of the methodogy with respect to t_R_, peak shape and signal intensity.

**Table S1** **POSITIVE ESI, Lipids contributing to the time related trajectory observed in PCA plot IV**

| **VIP** | **Retention Time** | **m/z** | **Neutral mass (Da)** | **Compound ID** | **Mass Error (ppm)** | **Max Fold Change** |
| --- | --- | --- | --- | --- | --- | --- |
| **1** | **4.55** | **920.7665** | **902.7326** | **TG(56:8)** | **-4.11** | **1.73** |
| **2** | **2.64** | **834.6000** | **833.5927** | **PC(40:6)** | **-0.85** | **1.62** |
| **3** | **4.43** | **973.7257** | **950.7365** | **TG(60:12)** | **0.14** | **2.53** |
| **4** | **2.28** | **758.5696** | **757.5623** | **PC(34:2)** | **0.21** | **1.56** |
| **5** | **1.97** | **806.5692** | **805.5619** | **PC(38:6)** | **-0.26** | **1.47** |

**Table S2:****POSITIVE ESI Lipids contributing to the time related trajectory observed in PCA plot PO**

| **VIP** | **Retention Time** | **m/z** | **Neutral mass (Da)** | **Lipids** | **Mass Error (ppm)** | **Max Fold Change** |
| --- | --- | --- | --- | --- | --- | --- |
| **1** | **0.55** | **496.3394** | **495.332** | **LPC(16:0)** | **-0.81** | **1.5** |
| **2** | **0.48** | **520.3389** | **519.332** | **LPC(18:2)** | **-1.73** | **2.2** |
| **3** | **0.46** | **544.3392** | **543.332** | **LPC(20:4)** | **-1.10** | **1.7** |
| **4** | **2.11** | **732.5540** | **731.554** | **PC(32:1)** | **0.27** | **-1.1** |
| **5** | **2.71** | **734.5699** | **769.584** | **PC(32:0)** | **0.68** | **0.5** |
| **7** | **2.95** | **786.5986** | **785.599** | **PC(36:2)** | **-2.67** | **-1.4** |
| **8** | **3.42** | **788.6167** | **787.609** | **PC(36:1)** | **0.38** | **-1.2** |

**Table S3: The variance in response of selected standards in the SPLASH LIPIDOMIX spiked into QC’s (n=19) used in the analysis of the mouse plasma samples**

**Lipid *m/z*  t_R_ (min) Mean SD %CV**

**intensity**

15:0-18:1(d7) PC 753.6137 2.41 405794 57891 14.3

15:0-18:1(d7) PE 711.5686 4.24 21724 1979 9.10

18:1(d7) Lyso PC 529.3995 0.55 26047 2812 10.8

15:0-18:1(d7) PS 755.5591 0.88 469.1 49.8 10.6

d18:1-18:1(d9) SM 738.6461 1.70 143.1 11.1 7.76

**Table S4:The Top 100 lipid features identified by OPLS-DA as being dysregulated**

**following the IV or PO administration of gefitinib**

**Negative Ion Data**

| **Lipid Feature** | **Polarity** | **Dose Route** | **p.ANOVA** | **FDR** |
| --- | --- | --- | --- | --- |
| 969.6088m/z | ESI- | IV | 5.33E-27 | 1.98E-24 |
| 689.5583m/z | ESI- | IV | 7.49E-26 | 2.08E-23 |
| 749.5790m/z | ESI- | IV | 2.63E-25 | 6.51E-23 |
| 708.4876m/z | ESI- | IV | 7.09E-25 | 1.58E-22 |
| 676.4720m/z | ESI- | IV | 1.39E-24 | 2.82E-22 |
| 653.4038m/z | ESI- | IV | 1.43E-22 | 2.27E-20 |
| 859.5478n | ESI- | IV | 7.18E-22 | 1.07E-19 |
| 874.5584m/z | ESI- | IV | 1.21E-21 | 1.68E-19 |
| 961.6031m/z | ESI- | IV | 9.21E-21 | 1.21E-18 |
| 656.4266n | ESI- | IV | 3.22E-20 | 3.98E-18 |
| 773.5819m/z | ESI- | IV | 3.01E-19 | 2.91E-17 |
| 766.5375m/z | ESI- | IV | 8.27E-19 | 7.08E-17 |
| 299.0242m/z | ESI- | IV | 1.09E-18 | 8.69E-17 |
| 970.6151n | ESI- | IV | 1.09E-18 | 8.69E-17 |
| 790.5389m/z | ESI- | IV | 2.59E-18 | 1.92E-16 |
| 611.4683m/z | ESI- | IV | 3.97E-18 | 2.85E-16 |
| 867.5527m/z | ESI- | IV | 5.73E-18 | 3.99E-16 |
| 373.0429m/z | ESI- | IV | 6.15E-18 | 4.15E-16 |
| 962.5414m/z | ESI- | IV | 8.63E-18 | 5.65E-16 |
| 609.4889m/z | ESI- | IV | 1.22E-17 | 7.30E-16 |
| 373.0428m/z | ESI- | IV | 1.26E-17 | 7.30E-16 |
| 814.5378m/z | ESI- | IV | 1.28E-17 | 7.30E-16 |
| 415.3193m/z | ESI- | IV | 2.62E-17 | 1.46E-15 |
| 810.5633m/z | ESI- | IV | 6.29E-17 | 3.18E-15 |
| 894.5458m/z | ESI- | IV | 1.46E-16 | 6.91E-15 |
| 669.4594m/z | ESI- | IV | 1.73E-16 | 7.86E-15 |
| 848.5430m/z | ESI- | IV | 2.78E-16 | 1.24E-14 |
| 812.5482m/z | ESI- | IV | 3.23E-16 | 1.40E-14 |
| 700.5998m/z | ESI- | IV | 3.55E-16 | 1.49E-14 |
| 781.6390n | ESI- | IV | 6.33E-16 | 2.61E-14 |
| 619.3444m/z | ESI- | IV | 6.57E-16 | 2.66E-14 |
| 779.6227n | ESI- | IV | 7.73E-16 | 3.07E-14 |
| 892.5308m/z | ESI- | IV | 1.19E-15 | 4.57E-14 |
| 913.5820m/z | ESI- | IV | 1.29E-15 | 4.85E-14 |
| 850.5609m/z | ESI- | IV | 1.70E-15 | 6.31E-14 |
| 800.5430m/z | ESI- | IV | 2.34E-15 | 8.55E-14 |
| 283.2411m/z | ESI- | IV | 2.39E-15 | 8.56E-14 |
| 960.5176m/z | ESI- | IV | 2.97E-15 | 1.05E-13 |
| 727.6189m/z | ESI- | IV | 3.12E-15 | 1.09E-13 |
| 821.4947m/z | ESI- | IV | 4.24E-15 | 1.43E-13 |
| 524.3332m/z | ESI- | IV | 4.29E-15 | 1.43E-13 |
| 354.2992m/z | ESI- | IV | 4.45E-15 | 1.46E-13 |
| 993.7537m/z | ESI- | IV | 4.91E-15 | 1.58E-13 |
| 688.5998m/z | ESI- | IV | 5.19E-15 | 1.65E-13 |
| 767.6233n | ESI- | IV | 5.98E-15 | 1.88E-13 |
| 919.5561n | ESI- | IV | 1.09E-14 | 3.29E-13 |
| 787.5740m/z | ESI- | IV | 1.10E-14 | 3.29E-13 |
| 367.2452m/z | ESI- | IV | 1.15E-14 | 3.38E-13 |
| 736.5180m/z | ESI- | IV | 1.21E-14 | 3.49E-13 |
| 760.4141m/z | ESI- | IV | 1.27E-14 | 3.62E-13 |
| 774.5274m/z | ESI- | IV | 1.39E-14 | 3.91E-13 |
| 756.4225m/z | ESI- | IV | 1.74E-14 | 4.85E-13 |
| 715.5739m/z | ESI- | IV | 1.87E-14 | 5.14E-13 |
| 987.3919m/z | ESI- | IV | 2.01E-14 | 5.46E-13 |
| 798.5040m/z | ESI- | IV | 2.07E-14 | 5.55E-13 |
| 922.5771m/z | ESI- | IV | 2.39E-14 | 6.26E-13 |
| 698.6285m/z | ESI- | IV | 2.55E-14 | 6.61E-13 |
| 744.4213m/z | ESI- | IV | 3.37E-14 | 8.62E-13 |
| 718.5376m/z | ESI- | IV | 3.86E-14 | 9.75E-13 |
| 804.5049m/z | ESI- | IV | 4.25E-14 | 1.06E-12 |
| 990.5643m/z | ESI- | IV | 5.38E-14 | 1.33E-12 |
| 764.5485m/z | ESI- | IV | 6.33E-14 | 1.53E-12 |
| 396.2158m/z | ESI- | IV | 7.47E-14 | 1.79E-12 |
| 551.3571m/z | ESI- | IV | 8.07E-14 | 1.91E-12 |
| 747.4438m/z | ESI- | IV | 8.44E-14 | 1.97E-12 |
| 788.5426m/z | ESI- | IV | 8.50E-14 | 1.97E-12 |
| 819.5497m/z | ESI- | IV | 8.71E-14 | 2.00E-12 |
| 775.5950m/z | ESI- | IV | 1.00E-13 | 2.25E-12 |
| 795.6370m/z | ESI- | IV | 1.27E-13 | 2.80E-12 |
| 919.4057m/z | ESI- | IV | 1.68E-13 | 3.67E-12 |
| 674.5843m/z | ESI- | IV | 2.23E-13 | 4.82E-12 |
| 577.3724m/z | ESI- | IV | 2.57E-13 | 5.50E-12 |
| 395.2446m/z | ESI- | IV | 2.67E-13 | 5.67E-12 |
| 412.2932n | ESI- | IV | 3.08E-13 | 6.46E-12 |
| 299.2570m/z | ESI- | IV | 3.15E-13 | 6.52E-12 |
| 824.5427m/z | ESI- | IV | 3.16E-13 | 6.52E-12 |
| 874.4211m/z | ESI- | IV | 3.24E-13 | 6.62E-12 |
| 635.5051m/z | ESI- | IV | 3.32E-13 | 6.72E-12 |
| 732.4232m/z | ESI- | IV | 3.54E-13 | 7.10E-12 |
| 419.2444m/z | ESI- | IV | 3.78E-13 | 7.50E-12 |
| 647.3758m/z | ESI- | IV | 3.97E-13 | 7.82E-12 |
| 824.5430m/z | ESI- | IV | 4.61E-13 | 8.92E-12 |
| 764.5220m/z | ESI- | IV | 4.96E-13 | 9.52E-12 |
| 493.0672m/z | ESI- | IV | 5.24E-13 | 9.97E-12 |
| 729.6346m/z | ESI- | IV | 5.44E-13 | 1.03E-11 |
| 650.4564m/z | ESI- | IV | 6.00E-13 | 1.12E-11 |
| 490.3283m/z | ESI- | IV | 6.03E-13 | 1.12E-11 |
| 957.4683n | ESI- | IV | 6.46E-13 | 1.19E-11 |
| 339.2117m/z | ESI- | IV | 6.89E-13 | 1.25E-11 |
| 691.1870m/z | ESI- | IV | 6.90E-13 | 1.25E-11 |
| 710.6274m/z | ESI- | IV | 7.02E-13 | 1.26E-11 |
| 451.3401m/z | ESI- | IV | 7.45E-13 | 1.33E-11 |
| 668.4096m/z | ESI- | IV | 7.56E-13 | 1.34E-11 |
| 549.2959m/z | ESI- | IV | 9.06E-13 | 1.59E-11 |
| 794.4758m/z | ESI- | IV | 9.50E-13 | 1.65E-11 |
| 715.6189m/z | ESI- | IV | 1.24E-12 | 2.13E-11 |
| 851.6265m/z | ESI- | IV | 1.36E-12 | 2.33E-11 |
| 814.5585m/z | ESI- | IV | 1.43E-12 | 2.42E-11 |
| 901.5421m/z | ESI- | IV | 1.55E-12 | 2.62E-11 |
| 990.6593m/z | ESI- | IV | 1.61E-12 | 2.70E-11 |
| 867.6787m/z | ESI- | PO | 7.85E-112 | 4.72E-108 |
| 860.6280m/z | ESI- | PO | 8.28E-105 | 2.49E-101 |
| 664.6231m/z | ESI- | PO | 3.36E-99 | 6.73E-96 |
| 923.7406m/z | ESI- | PO | 8.48E-94 | 1.27E-90 |
| 624.5923m/z | ESI- | PO | 4.55E-92 | 5.46E-89 |
| 582.5450m/z | ESI- | PO | 3.16E-89 | 3.16E-86 |
| 680.6168m/z | ESI- | PO | 7.09E-88 | 6.09E-85 |
| 652.6229m/z | ESI- | PO | 7.05E-81 | 5.30E-78 |
| 400.3780m/z | ESI- | PO | 6.67E-72 | 4.46E-69 |
| 664.6229m/z | ESI- | PO | 5.85E-66 | 3.51E-63 |
| 636.5817m/z | ESI- | PO | 2.95E-64 | 1.61E-61 |
| 372.3466m/z | ESI- | PO | 4.37E-64 | 2.19E-61 |
| 610.5761m/z | ESI- | PO | 3.22E-55 | 8.41E-53 |
| 638.6078m/z | ESI- | PO | 2.10E-52 | 5.05E-50 |
| 398.3620m/z | ESI- | PO | 1.70E-48 | 3.40E-46 |
| 666.6368m/z | ESI- | PO | 2.75E-46 | 5.33E-44 |
| 386.3637m/z | ESI- | PO | 2.63E-43 | 4.79E-41 |
| 906.6708m/z | ESI- | PO | 5.68E-42 | 1.00E-39 |
| 994.7221m/z | ESI- | PO | 3.35E-40 | 5.76E-38 |
| 862.6438m/z | ESI- | PO | 1.42E-39 | 2.38E-37 |
| 730.5706m/z | ESI- | PO | 4.30E-32 | 6.98E-30 |
| 950.6921m/z | ESI- | PO | 2.95E-31 | 4.67E-29 |
| 367.3345m/z | ESI- | PO | 2.63E-29 | 3.95E-27 |
| 730.5651m/z | ESI- | PO | 2.76E-29 | 4.04E-27 |
| 587.5088m/z | ESI- | PO | 1.85E-27 | 2.58E-25 |
| 368.4243m/z | ESI- | PO | 1.57E-26 | 2.03E-24 |
| 690.6034n | ESI- | PO | 2.05E-26 | 2.56E-24 |
| 367.3346m/z | ESI- | PO | 1.28E-25 | 1.57E-23 |
| 689.4955m/z | ESI- | PO | 2.78E-25 | 3.35E-23 |
| 639.6162n | ESI- | PO | 5.80E-24 | 6.58E-22 |
| 244.1900m/z | ESI- | PO | 2.67E-23 | 2.97E-21 |
| 634.5734m/z | ESI- | PO | 1.13E-22 | 1.21E-20 |
| 535.4321m/z | ESI- | PO | 7.15E-22 | 7.54E-20 |
| 642.5115m/z | ESI- | PO | 7.84E-21 | 8.13E-19 |
| 768.6699m/z | ESI- | PO | 1.42E-20 | 1.45E-18 |
| 722.6000n | ESI- | PO | 2.76E-20 | 2.76E-18 |
| 578.6600m/z | ESI- | PO | 4.10E-20 | 3.97E-18 |
| 686.5689m/z | ESI- | PO | 2.05E-19 | 1.87E-17 |
| 670.5677n | ESI- | PO | 2.18E-19 | 1.95E-17 |
| 334.3087m/z | ESI- | PO | 2.61E-19 | 2.27E-17 |
| 370.6540m/z | ESI- | PO | 3.19E-19 | 2.74E-17 |
| 665.6317n | ESI- | PO | 3.89E-19 | 3.30E-17 |
| 530.4002n | ESI- | PO | 1.50E-18 | 1.22E-16 |
| 626.6054m/z | ESI- | PO | 6.79E-18 | 5.30E-16 |
| 565.5207m/z | ESI- | PO | 7.59E-18 | 5.85E-16 |
| 680.5712n | ESI- | PO | 1.21E-17 | 9.12E-16 |
| 311.2933m/z | ESI- | PO | 1.45E-17 | 1.08E-15 |
| 668.6536m/z | ESI- | PO | 1.56E-17 | 1.14E-15 |
| 691.6385m/z | ESI- | PO | 3.14E-17 | 2.22E-15 |
| 522.5969m/z | ESI- | PO | 5.05E-17 | 3.49E-15 |
| 709.5504m/z | ESI- | PO | 1.12E-16 | 7.45E-15 |
| 733.5977n | ESI- | PO | 1.53E-16 | 1.01E-14 |
| 597.5029m/z | ESI- | PO | 2.64E-16 | 1.73E-14 |
| 660.5882m/z | ESI- | PO | 2.73E-16 | 1.76E-14 |
| 766.6658m/z | ESI- | PO | 3.32E-16 | 2.10E-14 |
| 578.5271n | ESI- | PO | 8.48E-16 | 5.25E-14 |
| 617.5849m/z | ESI- | PO | 9.78E-16 | 6.00E-14 |
| 694.5682n | ESI- | PO | 1.60E-15 | 9.60E-14 |
| 657.5811m/z | ESI- | PO | 2.12E-15 | 1.26E-13 |
| 367.3325m/z | ESI- | PO | 2.75E-15 | 1.62E-13 |
| 598.4888m/z | ESI- | PO | 3.03E-15 | 1.75E-13 |
| 782.6876m/z | ESI- | PO | 6.06E-15 | 3.40E-13 |
| 540.4833n | ESI- | PO | 7.22E-15 | 4.02E-13 |
| 653.6312n | ESI- | PO | 1.24E-14 | 6.76E-13 |
| 695.5046m/z | ESI- | PO | 1.50E-14 | 8.06E-13 |
| 978.8570m/z | ESI- | PO | 1.74E-14 | 9.25E-13 |
| 367.3319m/z | ESI- | PO | 2.12E-14 | 1.11E-12 |
| 683.6184n | ESI- | PO | 2.38E-14 | 1.23E-12 |
| 369.8036m/z | ESI- | PO | 3.62E-14 | 1.84E-12 |
| 383.3371m/z | ESI- | PO | 5.74E-14 | 2.90E-12 |
| 702.6169m/z | ESI- | PO | 6.01E-14 | 3.01E-12 |
| 713.5835m/z | ESI- | PO | 6.80E-14 | 3.38E-12 |
| 695.5749m/z | ESI- | PO | 7.83E-14 | 3.86E-12 |
| 998.7129n | ESI- | PO | 1.22E-13 | 5.94E-12 |
| 627.5325m/z | ESI- | PO | 1.37E-13 | 6.64E-12 |
| 686.6047m/z | ESI- | PO | 1.55E-13 | 7.38E-12 |
| 674.6029m/z | ESI- | PO | 1.59E-13 | 7.49E-12 |
| 584.6118m/z | ESI- | PO | 1.59E-13 | 7.49E-12 |
| 729.4820m/z | ESI- | PO | 1.61E-13 | 7.49E-12 |
| 942.5417m/z | ESI- | PO | 1.62E-13 | 7.49E-12 |
| 636.6052n | ESI- | PO | 2.11E-13 | 9.68E-12 |
| 957.7045m/z | ESI- | PO | 2.59E-13 | 1.18E-11 |
| 685.5298m/z | ESI- | PO | 2.62E-13 | 1.18E-11 |
| 996.7369n | ESI- | PO | 2.70E-13 | 1.20E-11 |
| 983.7271m/z | ESI- | PO | 2.95E-13 | 1.30E-11 |
| 645.6122n | ESI- | PO | 3.24E-13 | 1.42E-11 |
| 578.5117m/z | ESI- | PO | 3.34E-13 | 1.46E-11 |
| 685.6083m/z | ESI- | PO | 3.91E-13 | 1.69E-11 |
| 735.5686m/z | ESI- | PO | 3.95E-13 | 1.69E-11 |
| 677.4605m/z | ESI- | PO | 4.59E-13 | 1.96E-11 |
| 931.6980m/z | ESI- | PO | 4.68E-13 | 1.98E-11 |
| 284.3302m/z | ESI- | PO | 4.90E-13 | 2.05E-11 |
| 712.6013m/z | ESI- | PO | 5.77E-13 | 2.39E-11 |
| 528.3195m/z | ESI- | PO | 6.74E-13 | 2.74E-11 |
| 408.3078m/z | ESI- | PO | 6.76E-13 | 2.74E-11 |
| 869.5804n | ESI- | PO | 8.18E-13 | 3.30E-11 |
| 645.4723m/z | ESI- | PO | 9.03E-13 | 3.59E-11 |
| 933.7063m/z | ESI- | PO | 9.21E-13 | 3.64E-11 |
| 383.3290m/z | ESI- | PO | 9.64E-13 | 3.79E-11 |
| 551.5034m/z | ESI- | PO | 9.89E-13 | 3.86E-11 |

**Positive Ion Data**

| **Lipid Feature** | **Polarity** | **Dose Route** | **p.ANOVA** | **FDR** |
| --- | --- | --- | --- | --- |
| 669.5210m/z | ESI+ | IV | 7.32E-68 | 4.38E-64 |
| 782.6876m/z | ESI+ | IV | 1.38E-57 | 4.13E-54 |
| 659.5429m/z | ESI+ | IV | 9.95E-56 | 1.98E-52 |
| 678.5580n | ESI+ | IV | 5.66E-55 | 8.47E-52 |
| 766.7019m/z | ESI+ | IV | 4.28E-54 | 5.12E-51 |
| 782.6904m/z | ESI+ | IV | 2.69E-53 | 2.68E-50 |
| 678.5572n | ESI+ | IV | 4.27E-52 | 3.65E-49 |
| 752.6884m/z | ESI+ | IV | 1.07E-51 | 8.03E-49 |
| 662.5638n | ESI+ | IV | 3.11E-51 | 2.07E-48 |
| 687.5675m/z | ESI+ | IV | 1.22E-50 | 7.28E-48 |
| 715.5639m/z | ESI+ | IV | 2.08E-50 | 1.13E-47 |
| 383.3290m/z | ESI+ | IV | 3.31E-50 | 1.65E-47 |
| 682.6119m/z | ESI+ | IV | 4.71E-50 | 2.17E-47 |
| 687.5684m/z | ESI+ | IV | 5.96E-50 | 2.55E-47 |
| 780.6822m/z | ESI+ | IV | 7.28E-50 | 2.90E-47 |
| 310.2137n | ESI+ | IV | 1.03E-48 | 3.84E-46 |
| 699.5701m/z | ESI+ | IV | 1.11E-48 | 3.90E-46 |
| 680.5863n | ESI+ | IV | 5.64E-48 | 1.87E-45 |
| 741.5427m/z | ESI+ | IV | 1.23E-47 | 3.88E-45 |
| 383.3290m/z | ESI+ | IV | 1.66E-47 | 4.97E-45 |
| 294.2183n | ESI+ | IV | 3.76E-47 | 1.07E-44 |
| 750.6755m/z | ESI+ | IV | 2.58E-46 | 7.02E-44 |
| 383.3371m/z | ESI+ | IV | 3.01E-46 | 7.82E-44 |
| 766.6614m/z | ESI+ | IV | 2.26E-45 | 5.62E-43 |
| 727.5612m/z | ESI+ | IV | 2.56E-45 | 6.04E-43 |
| 764.6916m/z | ESI+ | IV | 2.62E-45 | 6.04E-43 |
| 709.5519m/z | ESI+ | IV | 9.52E-45 | 2.11E-42 |
| 766.6658m/z | ESI+ | IV | 1.23E-44 | 2.63E-42 |
| 367.3353m/z | ESI+ | IV | 5.92E-44 | 1.22E-41 |
| 949.7148m/z | ESI+ | IV | 4.20E-43 | 8.39E-41 |
| 709.5524m/z | ESI+ | IV | 1.10E-42 | 2.13E-40 |
| 725.5491m/z | ESI+ | IV | 1.32E-42 | 2.47E-40 |
| 680.5712n | ESI+ | IV | 2.87E-42 | 5.20E-40 |
| 718.5558n | ESI+ | IV | 5.22E-41 | 9.18E-39 |
| 697.5779m/z | ESI+ | IV | 7.43E-41 | 1.24E-38 |
| 683.5366m/z | ESI+ | IV | 7.46E-41 | 1.24E-38 |
| 733.5551m/z | ESI+ | IV | 8.49E-41 | 1.37E-38 |
| 766.7043m/z | ESI+ | IV | 9.43E-41 | 1.48E-38 |
| 774.6731m/z | ESI+ | IV | 1.75E-40 | 2.69E-38 |
| 719.5506m/z | ESI+ | IV | 3.56E-40 | 5.33E-38 |
| 725.5464m/z | ESI+ | IV | 5.92E-40 | 8.64E-38 |
| 666.6034m/z | ESI+ | IV | 1.34E-39 | 1.92E-37 |
| 694.6340m/z | ESI+ | IV | 1.40E-39 | 1.95E-37 |
| 733.5567m/z | ESI+ | IV | 1.50E-39 | 2.04E-37 |
| 337.2723m/z | ESI+ | IV | 1.82E-39 | 2.42E-37 |
| 798.6760m/z | ESI+ | IV | 1.99E-39 | 2.59E-37 |
| 743.5571m/z | ESI+ | IV | 2.72E-39 | 3.47E-37 |
| 661.5535m/z | ESI+ | IV | 3.02E-39 | 3.77E-37 |
| 881.2194m/z | ESI+ | IV | 5.10E-39 | 6.23E-37 |
| 740.5248m/z | ESI+ | IV | 5.90E-39 | 7.06E-37 |
| 752.6869m/z | ESI+ | IV | 7.91E-39 | 9.28E-37 |
| 638.5713m/z | ESI+ | IV | 1.17E-38 | 1.35E-36 |
| 686.5750n | ESI+ | IV | 1.84E-38 | 2.08E-36 |
| 734.6501m/z | ESI+ | IV | 1.96E-38 | 2.17E-36 |
| 667.5340m/z | ESI+ | IV | 2.63E-38 | 2.86E-36 |
| 778.5706n | ESI+ | IV | 4.05E-38 | 4.32E-36 |
| 719.5563m/z | ESI+ | IV | 4.52E-38 | 4.74E-36 |
| 746.5987m/z | ESI+ | IV | 1.13E-37 | 1.16E-35 |
| 930.8373m/z | ESI+ | IV | 1.39E-37 | 1.41E-35 |
| 1021.3376m/z | ESI+ | IV | 3.66E-37 | 3.65E-35 |
| 920.3093m/z | ESI+ | IV | 1.37E-36 | 1.35E-34 |
| 706.5946m/z | ESI+ | IV | 1.50E-36 | 1.44E-34 |
| 309.2781m/z | ESI+ | IV | 3.00E-36 | 2.85E-34 |
| 721.5902m/z | ESI+ | IV | 3.05E-36 | 2.85E-34 |
| 920.8581m/z | ESI+ | IV | 3.56E-36 | 3.28E-34 |
| 610.5401m/z | ESI+ | IV | 4.53E-36 | 4.11E-34 |
| 818.5814m/z | ESI+ | IV | 6.75E-36 | 6.02E-34 |
| 741.5413m/z | ESI+ | IV | 8.95E-36 | 7.88E-34 |
| 374.3414m/z | ESI+ | IV | 1.70E-35 | 1.48E-33 |
| 867.4758m/z | ESI+ | IV | 2.50E-35 | 2.14E-33 |
| 321.0492n | ESI+ | IV | 3.71E-35 | 3.13E-33 |
| 738.5573m/z | ESI+ | IV | 4.49E-35 | 3.73E-33 |
| 371.7497m/z | ESI+ | IV | 8.26E-35 | 6.77E-33 |
| 497.2807n | ESI+ | IV | 1.20E-34 | 9.70E-33 |
| 740.5247m/z | ESI+ | IV | 1.29E-34 | 1.02E-32 |
| 832.8700m/z | ESI+ | IV | 1.30E-34 | 1.02E-32 |
| 481.2196n | ESI+ | IV | 1.40E-34 | 1.09E-32 |
| 787.5399n | ESI+ | IV | 2.37E-34 | 1.82E-32 |
| 624.5688n | ESI+ | IV | 5.18E-34 | 3.92E-32 |
| 372.0434m/z | ESI+ | IV | 1.33E-33 | 9.98E-32 |
| 692.6015m/z | ESI+ | IV | 1.52E-33 | 1.13E-31 |
| 373.0138m/z | ESI+ | IV | 2.23E-33 | 1.62E-31 |
| 719.4626m/z | ESI+ | IV | 3.08E-33 | 2.22E-31 |
| 367.3346m/z | ESI+ | IV | 4.16E-33 | 2.96E-31 |
| 345.2364m/z | ESI+ | IV | 4.91E-33 | 3.46E-31 |
| 616.1765m/z | ESI+ | IV | 7.88E-33 | 5.49E-31 |
| 743.4650m/z | ESI+ | IV | 8.92E-33 | 6.14E-31 |
| 647.5588m/z | ESI+ | IV | 9.30E-33 | 6.32E-31 |
| 535.4321m/z | ESI+ | IV | 1.19E-32 | 8.01E-31 |
| 870.5230m/z | ESI+ | IV | 1.54E-32 | 1.03E-30 |
| 334.2842n | ESI+ | IV | 1.74E-32 | 1.14E-30 |
| 783.6542m/z | ESI+ | IV | 1.78E-32 | 1.16E-30 |
| 1110.8484m/z | ESI+ | IV | 2.78E-32 | 1.79E-30 |
| 367.3349m/z | ESI+ | IV | 4.47E-32 | 2.84E-30 |
| 682.6337m/z | ESI+ | IV | 6.92E-32 | 4.36E-30 |
| 656.5137n | ESI+ | IV | 9.13E-32 | 5.69E-30 |
| 1026.8479m/z | ESI+ | IV | 1.28E-31 | 7.90E-30 |
| 373.1468m/z | ESI+ | IV | 1.34E-31 | 8.20E-30 |
| 932.8595m/z | ESI+ | IV | 1.49E-31 | 9.01E-30 |
| 862.5089m/z | ESI+ | IV | 2.17E-31 | 1.30E-29 |
| 867.6787m/z | ESI+ | PO | 7.85E-112 | 4.72E-108 |
| 860.6280m/z | ESI+ | PO | 8.28E-105 | 2.49E-101 |
| 664.6231m/z | ESI+ | PO | 3.36E-99 | 6.73E-96 |
| 923.7406m/z | ESI+ | PO | 8.48E-94 | 1.27E-90 |
| 624.5923m/z | ESI+ | PO | 4.55E-92 | 5.46E-89 |
| 582.5450m/z | ESI+ | PO | 3.16E-89 | 3.16E-86 |
| 680.6168m/z | ESI+ | PO | 7.09E-88 | 6.09E-85 |
| 652.6229m/z | ESI+ | PO | 7.05E-81 | 5.30E-78 |
| 400.3780m/z | ESI+ | PO | 6.67E-72 | 4.46E-69 |
| 664.6229m/z | ESI+ | PO | 5.85E-66 | 3.51E-63 |
| 636.5817m/z | ESI+ | PO | 2.95E-64 | 1.61E-61 |
| 372.3466m/z | ESI+ | PO | 4.37E-64 | 2.19E-61 |
| 610.5761m/z | ESI+ | PO | 3.22E-55 | 8.41E-53 |
| 638.6078m/z | ESI+ | PO | 2.10E-52 | 5.05E-50 |
| 398.3620m/z | ESI+ | PO | 1.70E-48 | 3.40E-46 |
| 666.6368m/z | ESI+ | PO | 2.75E-46 | 5.33E-44 |
| 386.3637m/z | ESI+ | PO | 2.63E-43 | 4.79E-41 |
| 906.6708m/z | ESI+ | PO | 5.68E-42 | 1.00E-39 |
| 994.7221m/z | ESI+ | PO | 3.35E-40 | 5.76E-38 |
| 862.6438m/z | ESI+ | PO | 1.42E-39 | 2.38E-37 |
| 730.5706m/z | ESI+ | PO | 4.30E-32 | 6.98E-30 |
| 950.6921m/z | ESI+ | PO | 2.95E-31 | 4.67E-29 |
| 447.3742m/z | ESI+ | PO | 1.16E-29 | 1.78E-27 |
| 367.3345m/z | ESI+ | PO | 2.63E-29 | 3.95E-27 |
| 730.5651m/z | ESI+ | PO | 2.76E-29 | 4.04E-27 |
| 1076.7971m/z | ESI+ | PO | 5.35E-29 | 7.65E-27 |
| 587.5088m/z | ESI+ | PO | 1.85E-27 | 2.58E-25 |
| 294.1675m/z | ESI+ | PO | 5.39E-27 | 7.36E-25 |
| 340.3900m/z | ESI+ | PO | 8.34E-27 | 1.11E-24 |
| 368.4243m/z | ESI+ | PO | 1.57E-26 | 2.03E-24 |
| 313.1055m/z | ESI+ | PO | 1.59E-26 | 2.03E-24 |
| 690.6034n | ESI+ | PO | 2.05E-26 | 2.56E-24 |
| 367.3346m/z | ESI+ | PO | 1.28E-25 | 1.57E-23 |
| 689.4955m/z | ESI+ | PO | 2.78E-25 | 3.35E-23 |
| 367.3346m/z | ESI+ | PO | 6.86E-25 | 7.92E-23 |
| 639.6162n | ESI+ | PO | 5.80E-24 | 6.58E-22 |
| 244.1900m/z | ESI+ | PO | 2.67E-23 | 2.97E-21 |
| 634.5734m/z | ESI+ | PO | 1.13E-22 | 1.21E-20 |
| 535.4321m/z | ESI+ | PO | 7.15E-22 | 7.54E-20 |
| 642.5115m/z | ESI+ | PO | 7.84E-21 | 8.13E-19 |
| 768.6699m/z | ESI+ | PO | 1.42E-20 | 1.45E-18 |
| 722.6000n | ESI+ | PO | 2.76E-20 | 2.76E-18 |
| 443.1222m/z | ESI+ | PO | 3.96E-20 | 3.91E-18 |
| 578.6600m/z | ESI+ | PO | 4.10E-20 | 3.97E-18 |
| 494.5658m/z | ESI+ | PO | 5.19E-20 | 4.96E-18 |
| 1131.9314m/z | ESI+ | PO | 7.23E-20 | 6.79E-18 |
| 1155.9315m/z | ESI+ | PO | 8.76E-20 | 8.10E-18 |
| 686.5689m/z | ESI+ | PO | 2.05E-19 | 1.87E-17 |
| 670.5677n | ESI+ | PO | 2.18E-19 | 1.95E-17 |
| 334.3087m/z | ESI+ | PO | 2.61E-19 | 2.27E-17 |
| 370.6540m/z | ESI+ | PO | 3.19E-19 | 2.74E-17 |
| 665.6317n | ESI+ | PO | 3.89E-19 | 3.30E-17 |
| 226.1797m/z | ESI+ | PO | 5.36E-19 | 4.47E-17 |
| 313.3186m/z | ESI+ | PO | 1.19E-18 | 9.78E-17 |
| 530.4002n | ESI+ | PO | 1.50E-18 | 1.22E-16 |
| 1098.8306m/z | ESI+ | PO | 1.65E-18 | 1.32E-16 |
| 479.3704m/z | ESI+ | PO | 1.86E-18 | 1.47E-16 |
| 626.6054m/z | ESI+ | PO | 6.79E-18 | 5.30E-16 |
| 565.5207m/z | ESI+ | PO | 7.59E-18 | 5.85E-16 |
| 680.5712n | ESI+ | PO | 1.21E-17 | 9.12E-16 |
| 311.2933m/z | ESI+ | PO | 1.45E-17 | 1.08E-15 |
| 668.6536m/z | ESI+ | PO | 1.56E-17 | 1.14E-15 |
| 429.3719m/z | ESI+ | PO | 2.56E-17 | 1.83E-15 |
| 691.6385m/z | ESI+ | PO | 3.14E-17 | 2.22E-15 |
| 221.1742m/z | ESI+ | PO | 3.75E-17 | 2.62E-15 |
| 522.5969m/z | ESI+ | PO | 5.05E-17 | 3.49E-15 |
| 464.3139m/z | ESI+ | PO | 6.68E-17 | 4.56E-15 |
| 709.5504m/z | ESI+ | PO | 1.12E-16 | 7.45E-15 |
| 733.5977n | ESI+ | PO | 1.53E-16 | 1.01E-14 |
| 597.5029m/z | ESI+ | PO | 2.64E-16 | 1.73E-14 |
| 660.5882m/z | ESI+ | PO | 2.73E-16 | 1.76E-14 |
| 766.6658m/z | ESI+ | PO | 3.32E-16 | 2.10E-14 |
| 578.5271n | ESI+ | PO | 8.48E-16 | 5.25E-14 |
| 617.5849m/z | ESI+ | PO | 9.78E-16 | 6.00E-14 |
| 694.5682n | ESI+ | PO | 1.60E-15 | 9.60E-14 |
| 657.5811m/z | ESI+ | PO | 2.12E-15 | 1.26E-13 |
| 367.3325m/z | ESI+ | PO | 2.75E-15 | 1.62E-13 |
| 447.3457m/z | ESI+ | PO | 2.80E-15 | 1.64E-13 |
| 598.4888m/z | ESI+ | PO | 3.03E-15 | 1.75E-13 |
| 392.1031m/z | ESI+ | PO | 3.69E-15 | 2.11E-13 |
| 227.1876n | ESI+ | PO | 5.27E-15 | 2.99E-13 |
| 782.6876m/z | ESI+ | PO | 6.06E-15 | 3.40E-13 |
| 540.4833n | ESI+ | PO | 7.22E-15 | 4.02E-13 |
| 1022.7379n | ESI+ | PO | 8.31E-15 | 4.58E-13 |
| 653.6312n | ESI+ | PO | 1.24E-14 | 6.76E-13 |
| 429.3733m/z | ESI+ | PO | 1.33E-14 | 7.20E-13 |
| 695.5046m/z | ESI+ | PO | 1.50E-14 | 8.06E-13 |
| 978.8570m/z | ESI+ | PO | 1.74E-14 | 9.25E-13 |
| 367.3319m/z | ESI+ | PO | 2.12E-14 | 1.11E-12 |
| 683.6184n | ESI+ | PO | 2.38E-14 | 1.23E-12 |
| 1024.7527n | ESI+ | PO | 2.74E-14 | 1.41E-12 |
| 369.8036m/z | ESI+ | PO | 3.62E-14 | 1.84E-12 |
| 383.3371m/z | ESI+ | PO | 5.74E-14 | 2.90E-12 |
| 702.6169m/z | ESI+ | PO | 6.01E-14 | 3.01E-12 |
| 713.5835m/z | ESI+ | PO | 6.80E-14 | 3.38E-12 |
| 695.5749m/z | ESI+ | PO | 7.83E-14 | 3.86E-12 |
| 998.7129n | ESI+ | PO | 1.22E-13 | 5.94E-12 |
| 627.5325m/z | ESI+ | PO | 1.37E-13 | 6.64E-12 |
| 283.2625m/z | ESI+ | PO | 1.47E-13 | 7.05E-12 |
| 686.6047m/z | ESI+ | PO | 1.55E-13 | 7.38E-12 |
